# Surface Anchoring of the Kingella kingae Galactan Is Dependent on the Lipopolysaccharide O-Antigen

**DOI:** 10.1128/mbio.02295-22

**Published:** 2022-09-07

**Authors:** Nina R. Montoya, Eric A. Porsch, Vanessa L. Muñoz, Artur Muszyński, Jiri Vlach, David K. Hahn, Parastoo Azadi, Matthew Sherman, Hyojik Yang, Courtney E. Chandler, Robert K. Ernst, Joseph W. St. Geme

**Affiliations:** a University of Pennsylvania Perelman School of Medicine, Philadelphia, Pennsylvania, USA; b Department of Pediatrics, Children's Hospital of Philadelphia, Philadelphia, Pennsylvania, USA; c Complex Carbohydrate Research Center, University of Georgia, Athens, Georgia, USA; d University of Maryland—Baltimore School of Dentistry, Microbial Pathogenesis, Baltimore, Maryland, USA; New York University School of Medicine

**Keywords:** Gram-negative bacteria, lipopolysaccharide, exopolysaccharide, pathogenesis

## Abstract

Kingella kingae is a leading cause of bone and joint infections and other invasive diseases in young children. A key K. kingae virulence determinant is a secreted exopolysaccharide that mediates resistance to serum complement and neutrophils and is required for full pathogenicity. The K. kingae exopolysaccharide is a galactofuranose homopolymer called galactan and is encoded by the *pamABC* genes in the *pamABCDE* locus. In this study, we sought to define the mechanism by which galactan is tethered on the bacterial surface, a prerequisite for mediating evasion of host immune mechanisms. We found that the *pamD* and *pamE* genes encode glycosyltransferases and are required for synthesis of an atypical lipopolysaccharide (LPS) O-antigen. The LPS O-antigen in turn is required for anchoring of galactan, a novel mechanism for association of an exopolysaccharide with the bacterial surface.

## INTRODUCTION

Kingella kingae is a Gram-negative coccobacillus and is a common member of the oropharyngeal microbiota in children ages 6 months to 4 years, generally as a commensal organism. In recent years, PCR-based diagnostics revealed that K. kingae is also a leading cause of osteoarticular infections and other invasive diseases in young children, highlighting the need for a more detailed understanding of K. kingae pathogenicity (1–3). To reach sites of disease, K. kingae must initially colonize the upper respiratory tract, breach the respiratory epithelium to gain access to the bloodstream, and then survive in the bloodstream (3–6). Intravascular survival is driven by specific bacterial factors, including both a capsular polysaccharide and an exopolysaccharide (5, 7, 8). Our prototype strain of K. kingae, KK01, expresses an exopolysaccharide that is a galactofuranose homopolymer with the structure [→5)-β-Gal*f*-(1→]*_n_* and is termed PAM-galactan (9, 10). Bendaoud et al. identified a five-gene locus designated *pamABCDE* that is necessary for PAM-galactan production and found that only *pamABC* were required for synthesis of the galactofuranose homopolymer in Escherichia coli (10). In this system, PAM-galactan could be isolated from whole-cell sonicates but not the bacterial surface, suggesting that there may be other genes required for secretion. Initially, PAM-galactan was thought to contain abundant DNA and hence was termed poly-DNA-containing antiadhesive material extract (PAM extract). Subsequent work established that the galactofuranose polymer does not contain DNA (9, 10); thus, in this work we will use the term “galactan” to refer to the [→5)-β-Gal*f*-(1→]*_n_* homopolymer.

While the functions of bacterial exopolysaccharides have traditionally been attributed to processes such as biofilm formation, our work has identified roles for the K. kingae galactan exopolysaccharide in immune evasion. In particular, galactan has been shown to play a crucial role in intravascular survival by inhibiting complement-mediated killing and blocking neutrophil phagocytosis (5, 8). These novel functions have prompted us to pursue a more detailed understanding of the mechanism of galactan surface presentation and surface anchoring.

Early characterization of K. kingae galactan suggested that it is loosely tethered to the bacterial outer membrane through an unknown mechanism, as it could be readily identified in whole-bacteria surface washes with phosphate-buffered saline (PBS) as well as mild acid extracts of prewashed whole bacteria, a technique used to release the polysaccharide capsule from its lipid anchor in the outer membrane (9). An example of exopolysaccharide tethering to the membrane has been observed in E. coli, which contains a colanic acid (CA) exopolysaccharide that is dynamically converted between a lipopolysaccharide (LPS)-linked form, a secreted form, and a membrane-anchored form in response to metabolic stress (11, 12). Similarly, the enterobacterial common antigen (ECA) in *Enterobacteriaceae* is tethered to the membrane by either a lipid moiety or a covalent linkage to the lipid A-core oligosaccharide of the LPS (13–15).

LPS is a multifunctional glycolipid and is highly abundant in the outer membrane of Gram-negative bacteria. The functions of LPS are largely influenced by the structure, and there is significant heterogeneity in LPS composition across bacterial species, reflected both in structural diversity and in the genetic heterogeneity of LPS biosynthetic machinery. LPS is anchored in the membrane by lipid A, which is covalently linked to the core oligosaccharide. In bacteria with a smooth LPS, the core oligosaccharide is further connected to an O-antigen composed of repeating sugar units (16). LPS is crucial for membrane integrity and creates a selective permeability barrier, inhibiting the diffusion of small hydrophobic molecules, including detergents and antibiotics (16–18). Similarly, the length of the O-antigen and the array of covalent LPS modifications serve to restrict access of antibodies and complement proteins to the bacterial surface (19–22). In addition, lipid A, which is recognized by Toll-like receptor 4, is a primary driver of the host inflammatory response (23). Given the interface between LPS and the host immune system, LPS remodeling is often associated with immune evasion (24).

In this work, we identified homology between the *pamD* and *pamE* predicted products and LPS-modifying glycosyltransferases. Consistent with this homology, we demonstrated that the *pamD* and *pamE* gene products mediate synthesis of an atypical LPS O-antigen and are essential for surface anchoring of galactan. In addition, we provide evidence that galactan is attached to the LPS O-antigen, facilitating galactan-mediated evasion of immune mechanisms.

## RESULTS

### Surface extracts from *pamDE* mutants lack galactan material.

**FIG 1:**
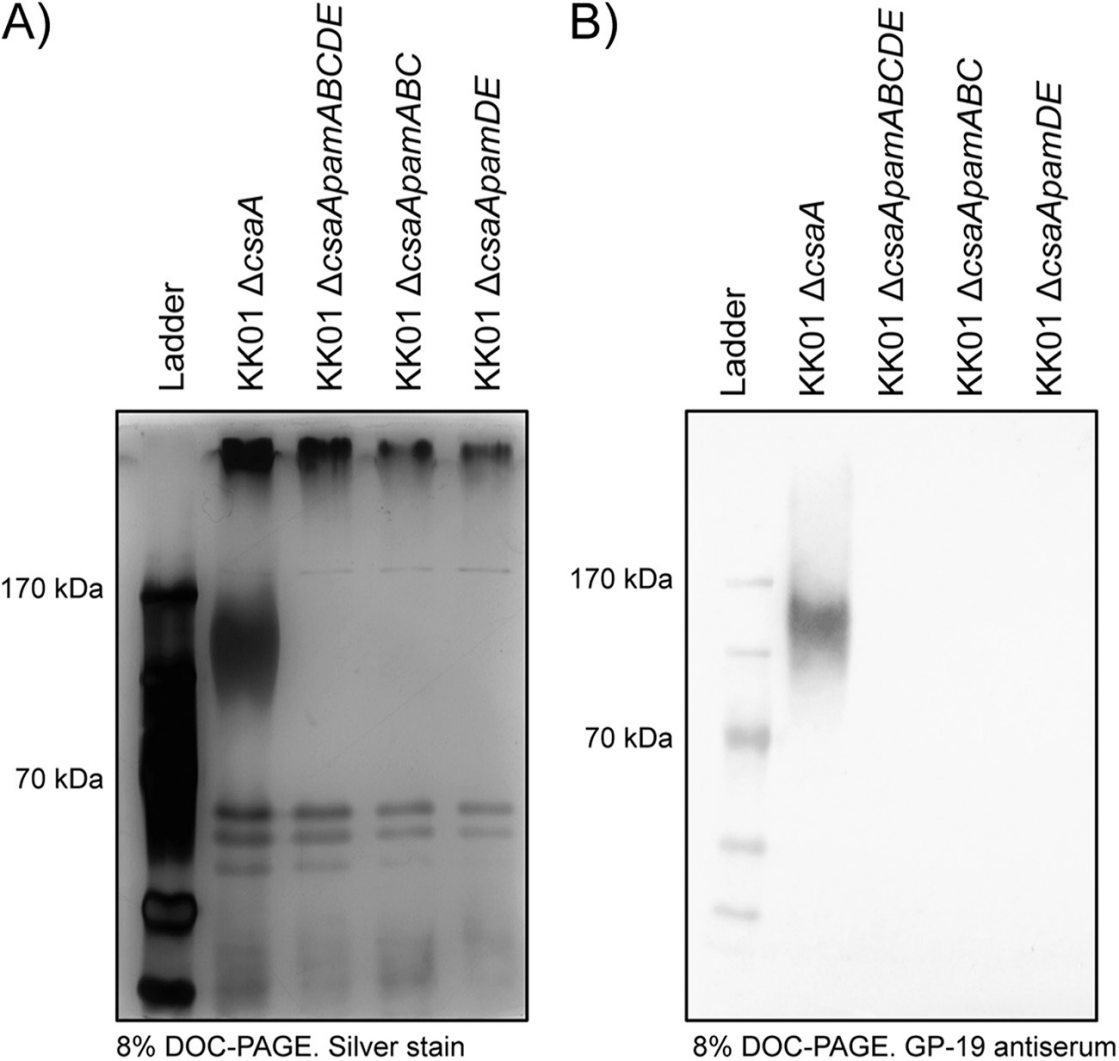
Galactan is not present in surface washes of Δ*csaApamDE* mutants. (A) Surface washes were isolated from K. kingae strains KK01 Δ*csaA*, KK01 Δ*csaApamABCDE*, KK01 Δ*csaApamABC*, and KK01 Δ*csaApamDE* by vortexing whole bacteria in PBS and concentrating the resulting material. Galactan in the samples was visualized by DOC-PAGE and silver stain. (B) The presence of galactan in bacterial surface washes was confirmed with detection by GP-19 antiserum, specific for the K. kingae galactan. Representative images are shown.

To assess the presence of galactan on the bacterial surface, we isolated surface washes from strains KK01 Δ*csaA* (lacking the capsule synthesis locus), KK01 Δ*csaApamABCDE* (a mutant lacking both the capsule synthesis locus and the full *pam* locus), KK01 Δ*csaApamABC* (a mutant lacking the capsule synthesis locus and the *pamABC* genes), and KK01 Δ*csaApamDE* (a mutant lacking the capsule synthesis locus and the *pamDE* genes). Strain KK01 Δ*csaA* was used as the parent strain for this analysis, to avoid contamination by the polysaccharide capsule. Surface washes were treated with DNase I, RNase A, and proteinase K and were then separated on a deoxycholic acid (DOC)-PAGE gel and stained with silver. As shown in Fig. 1A, the surface wash from strain KK01 Δ*csaA* contained a prominent, broad high-molecular-weight (HMW) silver-stained band. This band was absent in surface washes from strains KK01 Δ*csaApamABCDE*, KK01 Δ*csaApamABC*, and KK01 Δ*csaApamDE.* Based on previous work demonstrating that deletion of the *pamABCDE* genes results in the loss of galactan (9), we wondered whether the HMW band was galactan. To address this possibility, we generated a polyclonal antiserum against purified galactan recovered from the surface of KK01 Δ*csaA* (purity of 92.9% galactose as determined by gas chromatography-mass spectrometry [GC-MS]) (see Table S1 in the supplemental material). This antiserum (GP-19) was adsorbed with an acetone powder of strain KK01 Δ*csaApamABCDE* to remove nonspecific antibodies and was found to be reactive with whole-cell sonicates of E. coli JM109 harboring *pamABC* on a plasmid but not with whole-cell sonicates of E. coli JM109 harboring empty vector, confirming reactivity with galactan (see Fig. S1). Consistent with these results, Western immunoblot assays of surface washes performed with the GP-19 antiserum revealed reactivity with strain KK01 Δ*csaA* in the region corresponding to the size of the silver-stained material but no reactivity with strains KK01 Δ*csaApamABCDE*, KK01 Δ*csaApamABC*, or KK01 Δ*csaApamDE* (Fig. 1B), indicating that only strain KK01 Δ*csaA* contained galactan on the surface. These results demonstrated that the *pamD* and *pamE* genes are required for surface-associated galactan.

10.1128/mbio.02295-22.3 FIG S1 GP-19 antiserum is specific for the polymer synthesized by *pamABC* in E. coli JM109. (A) Galactan was detected by Western blotting with GP-19 in whole-cell sonicates from strains E. coli JM109 pTrc, JM109 pACYC, JM109 pTrc-*pamABC*, JM109 pACYC-*pamDE*, and JM109 pTrc-*pamABC* pACYC-*pamDE.* Representative image is shown. (B) Galactan was detected in whole-cell sonicates by ELISA with the GP-19 antiserum. Data are expressed as means ± SEM from three independent experiments. Download FIG S1, TIF file, 1.5 MB. Copyright © 2022 Montoya et al. 2022 Montoya et al. https://creativecommons.org/licenses/by/4.0/ This content is distributed under the terms of the Creative Commons Attribution 4.0 International license.

10.1128/mbio.02295-22.8 TABLE S1 Glycosyl composition of galactan prep for antibody production. Download Table S1, DOCX file, 0.01 MB. Copyright © 2022 Montoya et al. 2022 Montoya et al. https://creativecommons.org/licenses/by/4.0/ This content is distributed under the terms of the Creative Commons Attribution 4.0 International license.

### Wild-type K. kingae produces distinctive LPS molecules that migrate in a ladder pattern and as a HMW species and are lost in mutants lacking *pamD* and *pamE*.

**FIG 2:**
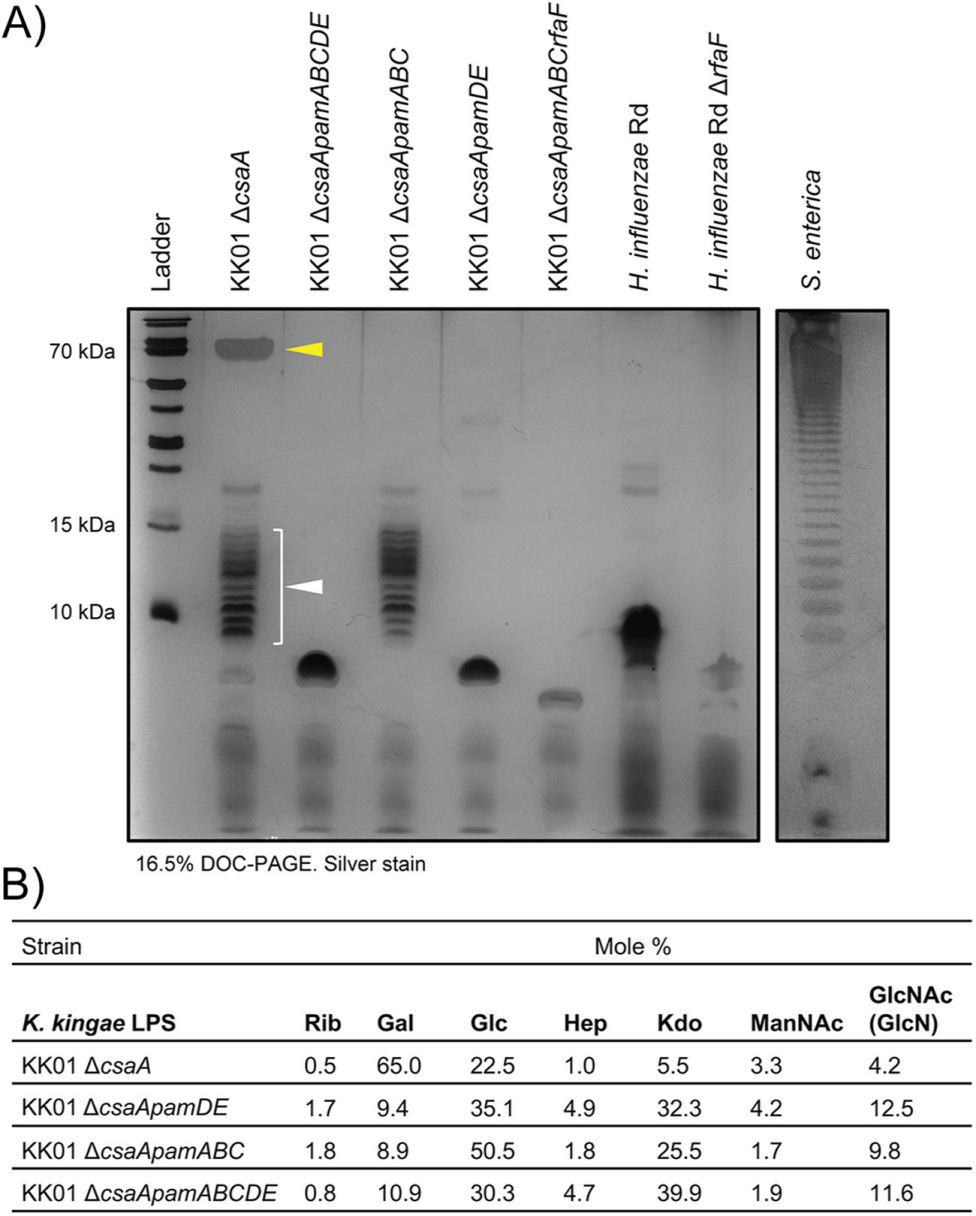
Wild-type K. kingae LPS is enriched in galactose and displays two modal clusters of LPS species that are selectively lost in Δc*saApamABC* and Δ*csaApamDE* mutants. (A) LPS was isolated from K. kingae strains KK01 Δ*csaA*, KK01 Δ*csaApamABCDE*, KK01 Δ*csaApamABC*, KK01 Δ*csaApamDE*, and KK01 Δ*csaApamABCrfaF* using a hot phenol extraction protocol. Rough LPS from strains H. influenzae Rd and H. influenzae Rd Δ*rfaF* and smooth LPS from S. enterica were run as controls. The KK01 Δ*csaA* LPS contains two modal clusters of LPS species, the low-molecular-weight (LMW) LPS, indicated by the white arrow, and the high-molecular-weight (HMW) LPS, indicated by the yellow arrow. A representative image is shown. (B) Comparative glycosyl composition analysis of LPS purified from the K. kingae KK01 Δ*csaA* parent strain and the KK01 Δ*csaApamDE*, KK01 Δ*csaApamABC*, and KK01 Δ*csaApamABCDE* mutants. Rib, ribose; Gal, galactose; Glc, glucose; Hep, heptose; Kdo, 3-deoxy-d-*manno*-octulosonic acid; ManNAc, *N*-acetylmannosamine; GlcNAc, *N*-acetylglucosamine; GlcN, glucosamine present in lipid A was converted to GlcNAc during the re-N-acetylation step of the chemical derivatization.

Homology analysis revealed that the *pamD* and *pamE* gene products share significant homology with GTB superfamily glycosyltransferases and family 25 LPS-modifying glycosyltransferases, respectively. To explore the possible role of the *pam* genes in biosynthesis of the K. kingae LPS, we compared the migration patterns of LPS samples prepared from strains KK01 Δ*csaA*, KK01 Δ*csaApamABCDE*, KK01 Δ*csaApamABC*, and KK01 Δ*csaApamDE* by using a hot phenol-based method; samples were separated on a DOC-PAGE gel and stained with silver. As shown in Fig. 2A, strain KK01 Δ*csaA* produced LPS that migrated as two distinct modal clusters, including a discrete high-molecular-weight species at the top of the gel (designated HMW LPS, yellow arrow) and a low-molecular-weight ladder of distinct bands (designated LMW LPS, white arrow and bracket). The LMW LPS was distinct from the lipooligosaccharide (LOS) produced by Haemophilus influenzae and was notably larger than the LOS truncated after the second heptose residue produced by an H. influenzae Δ*rfaF* mutant (25). Although distinct from a typical O-antigen produced by Salmonella enterica serovar Enterica, the observed ladder pattern suggested that the KK01 Δ*csaA* LMW LPS contains a repeating unit resembling O-antigens in other organisms (26, 27).

The KK01 Δ*csaApamABC* mutant lacked HMW LPS, and the KK01 Δ*csaApamDE* mutant lacked both LMW LPS and HMW LPS and displayed only a truncated LPS species, suggesting that the *pamD* and *pamE* genes are required for production of a repeating sugar unit. Of note, the KK01 Δ*csaApamDE* LPS was larger than the LOS produced by a KK01Δ*csaApamABCrfaF* mutant. Homology analysis suggested that the K. kingae *rfaF* gene encodes a lipooligosaccharide heptosyltransferase II, responsible for adding the second heptose residue onto the growing LOS. Deletion of the K. kingae *rfaF* gene resulted in a low-molecular-weight LOS molecule similar in size to the LOS produced by H. influenzae Δ*rfaF.* The KK01 Δ*csaApamABCrfaF* LOS was smaller than the truncated LOS produced by KK01 Δ*csaApamDE*, suggesting that KK01 Δ*csaApamDE* retains residues of the core oligosaccharide.

These results demonstrated that K. kingae produces two modal clusters of LPS species: (i) LMW LPS, composed of lipid A, core oligosaccharide, and an atypical O-antigen comprised of a repeating unit and adopting a range of sizes and (ii) HMW LPS, composed of lipid A, core oligosaccharide, an atypical O-antigen, and an additional large polysaccharide modification that may be attached to the O-antigen. These results also established that the *pamDE* genes are required for production of LMW LPS and that the complete *pamABCDE* locus is required for production of HMW LPS.

### The K. kingae LPS is primarily composed of galactose.

To determine the carbohydrate composition of the LPS molecules produced by KK01 Δ*csaA* and mutants lacking specific *pam* genes, we performed glycosyl composition analysis of purified LPS samples from these strains. This analysis revealed that galactose was the most abundant monosaccharide in KK01 Δ*csaA* LPS (Fig. 2B). In strains KK01 Δ*csaApamABCDE*, KK01 Δ*csaApamABC*, and KK01 Δ*csaApamDE*, the amount of galactose was markedly reduced, while the relative amounts of glucose, heptose, and 3-deoxy-d-manno-octulosonic acid (Kdo) were increased, likely reflecting a shortening of the polysaccharide attached to lipid A. The compositional analysis was consistent with the loss of galactose from HMW LPS in KK01 Δ*csaApamABC* and the loss of both LMW LPS and HMW LPS in strains KK01 Δ*csaApamABCDE* and KK01 Δ*csaApamDE.* These results established that K. kingae HMW LPS contains a large galactose polysaccharide, dependent on all the *pam* genes. Given our understanding of the role of *pamABC* in the biosynthesis of galactan, these data suggest that the presence of galactose in HMW LPS may result from anchoring of galactan to the LMW LPS atypical O-antigen.

### HMW LPS species react with antigalactan antiserum.

**FIG 3:**
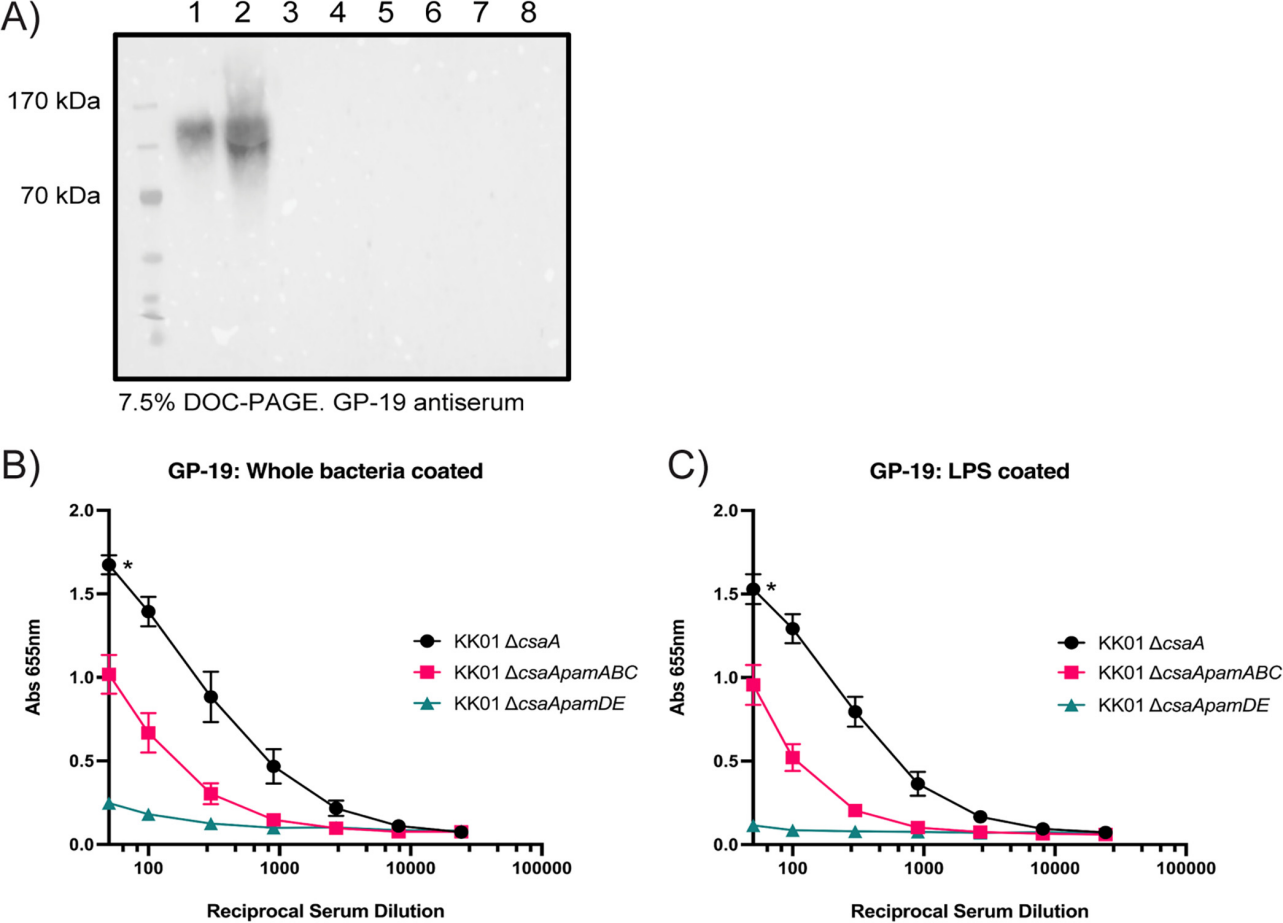
LPS from the Δ*pamDE* mutant does not react with GP-19 galactan antiserum. (A) LPS samples (odd lanes) and surface washes (even lanes) from K. kingae strains KK01 Δ*csaA* (lanes 1 and 2), Δ*csaApamABCDE* (lanes 3 and 4), Δ*csaApamABC* (lanes 5 and 6), and Δ*csaApamDE* (lanes 7 and 8) were separated by DOC-PAGE, and galactan was detected with GP-19 antiserum. A representative image is shown. (B and C) Galactan was detected in purified LPS samples (B) and in whole bacteria (C) with GP-19 antiserum by ELISA. Three biological replicates were performed. Data are expressed as means ± standard errors of the means (SEM) from three independent experiments. Statistical significance was determined by 2-way analysis of variance (ANOVA) with Tukey’s correction for multiple comparisons (all comparisons at a 1:50 serum dilution). *, *P *< 0.05.

To determine if the galactose component of HMW LPS was galactan, we used the GP-19 antiserum to detect galactan material in purified LPS samples from strains KK01 Δ*csaA*, KK01 Δ*csaApamABCDE*, KK01 Δ*csaApamABC*, and KK01 Δ*csaApamDE*. LPS samples were resolved on a DOC-PAGE gel and examined by Western immunoblotting with GP-19. As shown in Fig. 3, reactivity was observed with LPS from strain KK01 Δ*csaA* but not from strain KK01 Δ*csaApamABCDE* or strain KK01 Δ*csaApamABC*, indicating the presence of galactan in HMW LPS and highlighting the essential role of the *pamABC* genes in galactan biosynthesis. Reactivity was also lacking with LPS from strain KK01 Δ*csaApamDE*, consistent with the conclusion that the *pamD* and *pamE* genes are required for the presence of galactan in HMW LPS. In parallel experiments, we coated enzyme-linked immunosorbent assay (ELISA) plates with purified LPS samples and examined these plates with the GP-19 antiserum. Like the results observed by Western blotting, significant reactivity was detected with LPS from strain KK01 Δ*csaA* (Fig. 3B). These results indicated that galactan copurifies with HMW LPS.

In addition, the ELISA data revealed that plates coated with LPS from strain KK01 Δ*csaApamABC* generated an intermediate level of reactivity with GP-19 antiserum (Fig. 3B). At a 1:50 serum dilution, reactivity with strain KK01 Δ*csaApamABC* was significantly different than the reactivities of both strain KK01 Δ*csaA* and KK01 Δ*csaApamDE.* Because reactivity was negligible in plates coated with LPS from strain KK01 Δ*csaApamDE* (Fig. 3B), we posited that the intermediate reactivity observed with LPS from KK01 Δ*csaApamABC* was specifically generated against epitopes in LMW LPS requiring the *pamD* and *pamE* gene products, suggesting that the galactan sample used for antibody production also contained LPS. Similar trends were observed when ELISA plates were coated with whole bacteria (Fig. 3C).

### HMW LPS can be separated from LMW LPS by size exclusion and is enriched in galactofuranose.

**FIG 4:**
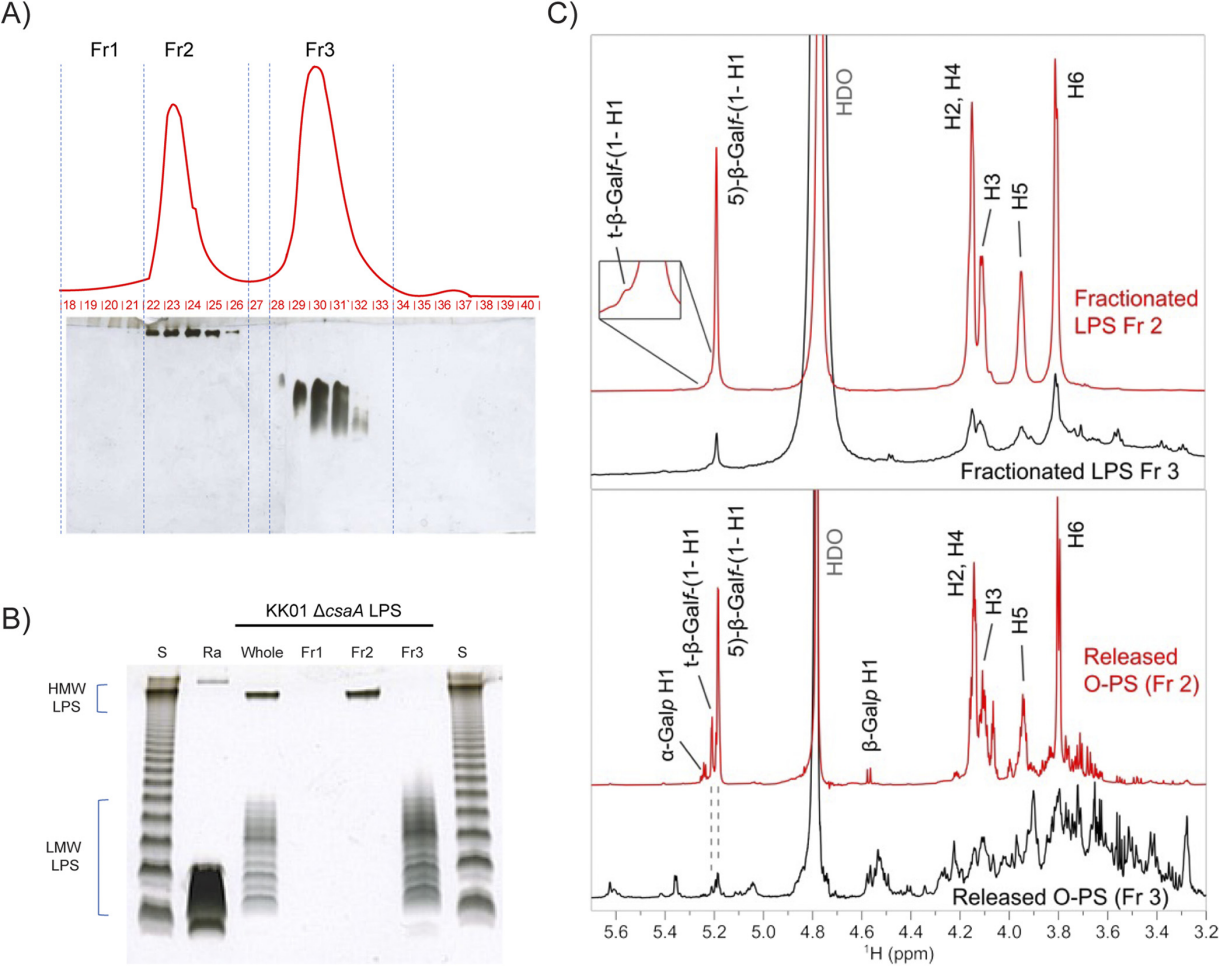
Gal*f* residues are predominantly found in HMW LPS species. (A) Size exclusion chromatography of K. kingae KK01 Δ*csaA* intact (whole) LPS. The LPS was resolved on a Superdex 75 column, under dissociative conditions, in the presence of deoxycholate. Fractions 1 to 3 (Fr1 to Fr3) were pooled, based on the refractive index (RI) response (red line) combined with the electrophoretic profile of all subfractions (fractions 19 to 40), resolved by DOC-PAGE and silver stained. (B) Comparative DOC-PAGE analysis of KK01 Δ*csaA* intact LPS (whole) and the same LPS fractionated on a Superdex 75 column (Fr1 to -3). Lane S, S. enterica serovar Minnesota (S-strain) LPS; lane Ra, *E. coli* EH100 LPS (Ra mutant) (both were used as controls). Each lane was loaded with 1 μg of the sample. (C) ^1^H NMR spectra of the fractionated LPS (Fr2 and Fr3) and the corresponding released O-PS material. The signals of the major Gal*f* polymer are labeled; for simplicity, the ring signals of 5)-β-Gal*f*-(1→ are marked only as H2 to H6. The released O-PS Fr2 contained free Gal*p* that likely formed from the acid-labile Gal*f* during LPS hydrolysis.

To establish definitively that the galactose component of K. kingae HMW LPS is identical to galactan {[→5)-β-Gal*f*-(1→]*_n_*}, we separated the HMW LPS species from the LMW LPS ladder using size exclusion chromatography (SEC) under dissociative conditions in the presence of a deoxycholic acid detergent. The SEC yielded two significant fractions corresponding to HMW LPS and LMW LPS (designated Fr2 and Fr3, respectively), confirmed by analysis of the individual subfractions (Fr2 [subfractions 22 and 23] and Fr3 [subfractions 28 to 33]) via DOC-PAGE (Fig. 4A). A comparative DOC-PAGE analysis of the pooled HMW LPS (Fr2) and LMW LPS (Fr3) with the “whole” unseparated LPS demonstrated successful isolation of HMW LPS from LMW LPS (Fig. 4B, compare lanes labeled Fr2, Fr3, and whole). Rough LPS from E. coli Ra and smooth LPS from S. enterica were used as controls.

The enriched HMW LPS and LMW LPS fractions (Fr2 and Fr3, respectively), as well as the total polysaccharide portion of LPS (O-PS), including core oligosaccharide, O-antigen, and galactan released from each fraction by mild acid hydrolysis, were then analyzed by nuclear magnetic resonance (NMR) spectroscopy. The ^1^H spectrum of HMW LPS (Fr2) contained dominant signals that corresponded to the →5)-β-Gal*f*-(1→ galactan polymer (Fig. 4C, top). The identity of the signals was confirmed by a heteronuclear single quantum coherence (HSQC) fingerprint (see Fig. S2) that matched with the data reported previously for galactan (9) as well as our data obtained on the released O-PS material (see below). The broadened signal appearance and low-intensity signal of the terminal β-Gal*f*-(1→ group were consistent with the high molecular weight of the polymer. The ^1^H spectrum of LMW LPS (Fr3) also contained predominantly the same carbohydrate signals as the HMW LPS spectrum (Fig. 4C, top), suggesting the presence of galactan in the LMW LPS. The broader appearance of the LMW LPS galactan signals was likely due to the formation of micelles, which was more pronounced in LMW LPS compared to HMW LPS. ^1^H NMR spectra of the polysaccharides released by acidic hydrolysis from the fractionated LPS are shown in Fig. 4C (bottom). The spectrum of the O-PS released from the HMW LPS contained predominantly signals of the galactan, which was identified using two-dimensional (2D)-NMR spectroscopy (see Fig. S3 and Table S2). Compared to intact LPS, the released O-PS contained a significantly higher proportion of the terminal β-Gal*f*-(1→ group, suggesting a reduced molecular weight of the polymer. LPS hydrolysis likely resulted in partial degradation of the highly acid-labile Gal*f* polymer, consistent with the presence of a small amount of free Gal*p* (Fig. 4C, bottom). The spectrum of the O-PS released from the LMW LPS contained signals of galactan as well as numerous additional signals likely due to O-antigen and core oligosaccharide residues. Overall, the NMR results supported the conclusion that the K. kingae HMW LPS contains a large →5)-β-Gal*f*-(1→ galactan polymer tethered to the LMW LPS glycolipid region. The LMW LPS contains a relatively small amount of short galactan polymers. This analysis did not allow us to identify a direct covalent linkage between the LMW LPS and galactan, which was likely influenced by the susceptibility of this glycosidic linkage to acid hydrolysis.

10.1128/mbio.02295-22.4 FIG S2 ^1^H and ^1^H,^13^C-HSQC NMR spectra of LPS Fr2. The HSQC signals of HMW LPS from Fr2 matched the galactan signals reported by Starr *et al*., 2013, as well as the signals of the galactan identified in the released carbohydrate material from Fr2 (see Fig. S3 and Table S2). Download FIG S2, TIF file, 0.1 MB. Copyright © 2022 Montoya et al. 2022 Montoya et al. https://creativecommons.org/licenses/by/4.0/ This content is distributed under the terms of the Creative Commons Attribution 4.0 International license.

10.1128/mbio.02295-22.5 FIG S3 2D NMR spectra of the carbohydrate material released from HMW LPS Fr2. The HSQC signals of the carbohydrate released from HMW LPS Fr2 were assigned with the aid of COSY, TOCSY, NOESY, and HMBC NMR spectra. The partial NOESY and HMBC spectra are shown along the axes of the HSQC spectrum to illustrate signals instrumental in the assignment of the galactan resonances as well as identification of interresidual correlations (marked in bold). Download FIG S3, TIF file, 0.1 MB. Copyright © 2022 Montoya et al. 2022 Montoya et al. https://creativecommons.org/licenses/by/4.0/ This content is distributed under the terms of the Creative Commons Attribution 4.0 International license.

10.1128/mbio.02295-22.9 TABLE S2 ^1^H and ^13^C chemical shift assignments of galactan released from HMW LPS Fr2. Download Table S2, DOCX file, 0.01 MB. Copyright © 2022 Montoya et al. 2022 Montoya et al. https://creativecommons.org/licenses/by/4.0/ This content is distributed under the terms of the Creative Commons Attribution 4.0 International license.

To determine if the KK01 Δ*csaApamABC* LPS contains any Gal*f* signals, we performed a comparative NMR analysis. The profile of the anomeric signals in ^1^H NMR spectrum of the total, unfractionated O-PS material released from KK01 Δ*csaApamABC* LMW LPS was very similar to that seen in the KK01 Δ*csaA* LMW O-PS Fr3 (see Fig. S4A), suggesting similar core and O-antigen structures. Using HSQC, we confirmed that the characteristic Gal*f* signals seen in the KK01 Δ*csaA* LMW O-PS were absent from the spectrum of the Δ*csaApamABC* O-PS (see Fig. S4B), suggesting that the galactose detected in its LPS (Fig. 2B) is in the pyranose form. Given the similarity of the LMW LPS species from KK01 Δ*csaA* and KK01 Δ*csaApamABC*, it is important to note that in the absence of the *pamABC* genes, the LMW LPS is devoid of Gal*f*. Combined, the NMR and the immunochemical analyses showed that the galactan polymer is not part of the KK01 Δ*csaApamABC* LPS and that *pamABC* are essential for incorporation of Gal*f* into the O-PS portion of LPS.

10.1128/mbio.02295-22.6 FIG S4 Gal*f* is not present in the KK01 *ΔcsaApamABC* LPS. (A) Comparison of a partial anomeric region of ^1^H NMR spectra of the KK01 Δ*csaA* released LMW O-PS (from LMW LPS Fr3, in black) and the total, unfractionated O-PS released from KK01 *ΔcsaApamABC* LPS (in red). The signals of Gal*f* H1, present in the KK01 *ΔcsaA* O-PS, are absent from the KK01 *ΔcsaApamABC* O-PS. (B) Comparison of the whole anomeric region of ^1^H,^13^C-HSQC NMR spectra of the above O-PS material from the two mutants. Green rectangle highlights the positions of the anomeric Gal*f* signals that are not present in the KK01 *ΔcsaApamABC* O-PS spectrum. The corresponding regions of the ^1^H spectra are shown above the HSQC spectra. Download FIG S4, TIF file, 1.1 MB. Copyright © 2022 Montoya et al. 2022 Montoya et al. https://creativecommons.org/licenses/by/4.0/ This content is distributed under the terms of the Creative Commons Attribution 4.0 International license.

### HMW LPS and LMW LPS species are anchored to the same lipid A molecule.

**FIG 5:**
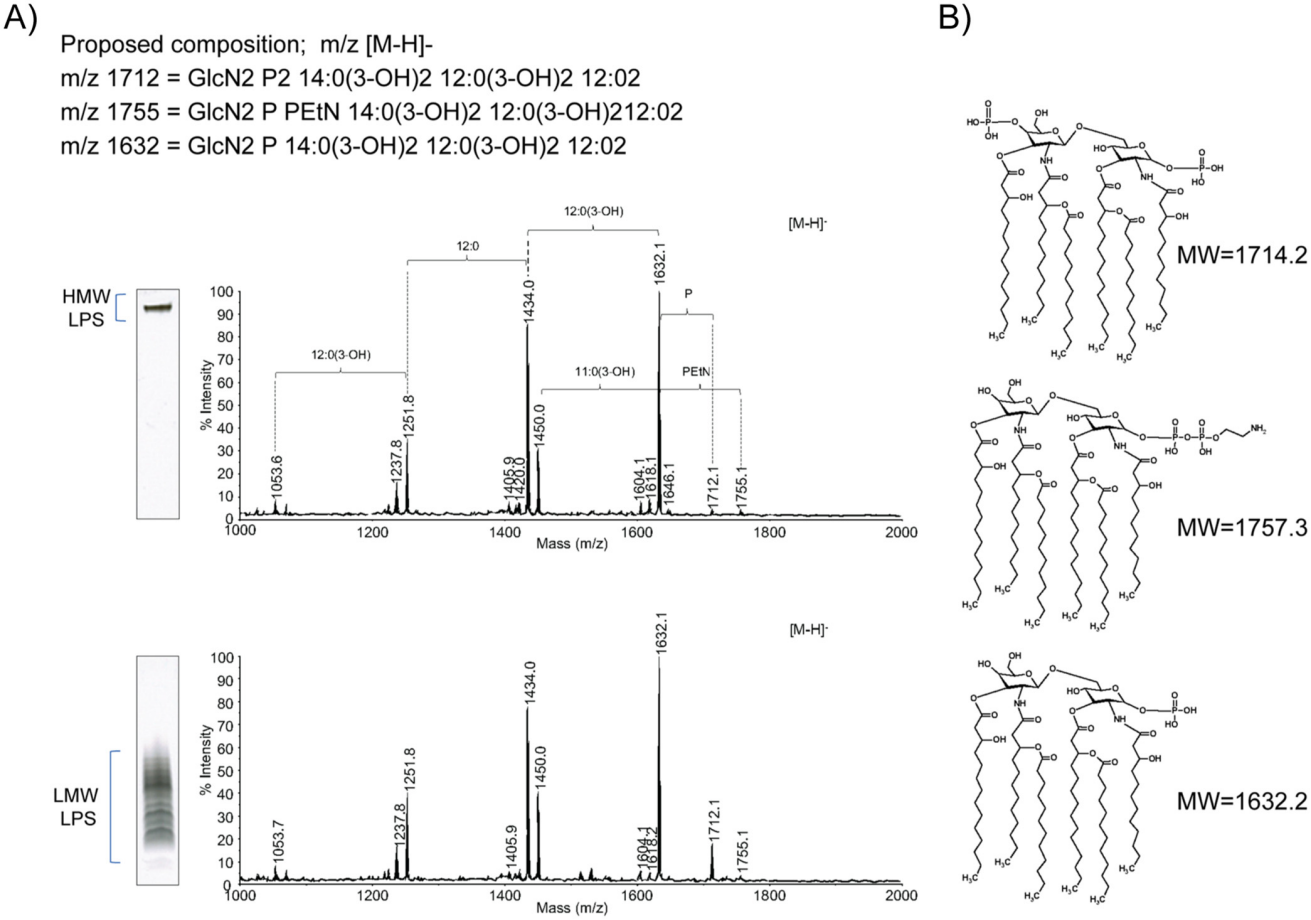
K. kingae lipid A can be identified in both HMW and LMW LPS clusters. (A) MALDI-TOF MS analysis of the lipid A recovered from the HMW LPS from KK01 Δ*csaA* (Fr2) (top spectrum) and from LMW LPS of KK01 Δ*csaA* (Fr3) (bottom spectrum) and proposed lipid A compositions. The spectra were acquired in the negative reflectron ionization mode. The DOC-PAGE insets show the HMW (Fr2) and LMW (Fr 3) LPS. (B) Proposed structures of lipid A corresponding to the selected [M-H]^−^ ions observed in the MALDI-TOF MS shown in panel A.

In the absence of direct identification of a covalent linkage between LMW LPS and galactan, we sought to determine whether HMW LPS and LMW LPS were both anchored to the same lipid A molecule, recognizing that lipid A is the membrane-embedded structure to which all other components of LPS are sequentially attached. We hypothesized that if the HMW LPS was composed of LMW LPS with a galactan modification, we would detect K. kingae lipid A in both the HMW LPS and the LMW LPS fractions. Matrix-assisted laser desorption ionization–time of flight mass spectrometry (MALDI-TOF MS) analysis of the lipid A released from HMW LPS and LMW LPS after hard acid hydrolysis showed that both fractions share identical MS lipid A profiles (Fig. 5A, Fr2 [top spectrum] versus Fr3 [lower spectrum]). A major detected [M-H]^−^ ion at *m/z* 1632.1 could be attributed to a *mono-*phosphoryl-hexa-acyl lipid A species consisting of (GlcN)_2_,P,(14:0(3-OH))_2_,(12:0(3-OH))_2_,(12:0)_2_. We also detected a minor ion at *m/z* 1712.1 representing *bis*-phosphoryl-hexa-acyl lipid A, consisting of (GlcN)_2_,P_2_,(14:0(3-OH))_2_,(12:0(3-OH))_2_,(12:0)_2_. The low-intensity ion at *m/z* 1755.1 could be attributed to the *mono*-phosphoryl-hexa-acyl lipid A substituted with phosphoethanolamine (PEtN) and containing (GlcN)_2_,P,PEtN(14:0(3-OH))_2_,(12:0(3-OH))_2_,(12:0)_2_. The relatively lower intensities of [M-H]^−^ ions corresponding to the *bis*-phosphoryl or PEtN-substituted lipid A in the MS analysis were likely caused by a susceptibility of these groups to 1% acetic acid during hydrolysis. In addition to these ions, both lipid A fractions resolved signals at *m/z* 1434.0 [loss of 12:0(3-OH) from the main observed structure (*m/z* 1632.1)], *m/z* 1450.0 [loss of 11:0(3-OH) from the main structure], *m/z* 1251.8 [loss of 12:0(3-OH) and 12:0 from the main structure], and *m/z* 1053.6 [loss of 12:0(3-OH)_2_ and 12:0 from the main structure]. All these detected ions, together with the finding of 12:0(3-OH), 14:0(3-OH), 11:0(3-OH), 12:0 in the composition analysis of KK01 Δ*csaA*, supported the proposed lipid A structures in Fig. 5B.

To better characterize the general structure of the K. kingae strain KK01 lipid A from unfractionated LPS, we used a milder lipid A hydrolytic condition on whole bacteria and carried out tandem MS (MS^2^) to confirm the structure of this lipid A molecule. Figure S5A shows the mass spectrum obtained from a bacterial solution of KK01 Δ*csaA* after fast lipid analysis technique (FLAT) processing (MS^1^) (28). Using the precursor ions at *m/z* 1712.12 and 1835.13, the *m/z* difference of 123.01 indicated that the lipid A has a PEtN modification (see Fig. S5A). MS^2^ was used to confirm the structure. We noted key bond cleavages and fragment ions generated by collision-induced dissociation and FLAT^n^ processing, and the indicated positions corresponding to each cleavage are shown in the accompanying structures (see Fig. S5C and D). For example, the fragment ion generated by the loss of H_3_PO_4_ from K. kingae lipid A corresponded to an ion at *m/z* 1614.14 (theoretical value) and was labeled B_2_ according to accepted nomenclature (29, 30). As expected, many other neutral losses were observed, including acyl chains from ester bonds and cross-ring cleavages. Multiple losses of acyl chains from ester bonds, such as *m/z* 1397.97 (B_2_ with 3′α), 1199.81 (B_2_ with 3′α and 3′β), and 999.63 (B_2_ with 3′α, 3 β, and 2’ ε) are shown in Fig. S5C. The cross-ring cleavage produced ions at *m/z* 690.40 corresponding to ^0,4^A_2_ plus 3′α. These selective dissociations of acyl chains from ester bonds and cross-ring cleavage of the backbone were used to deduce the locations of additional acylation groups and modifications of acyl chains. Furthermore, the modification site of lipid A by PEtN was characterized using FLAT^n^. The characteristic ion at *m/z* 987.57 represents the 1-phosphate group modification with PEtN, generated from Y_1_.

10.1128/mbio.02295-22.7 FIG S5 Biochemical characterization of K. kingae lipid A. (A) MALDI MS obtained in negative ion mode from bacterial solution of K. kingae Δ*csaA* after FLAT processing. Major species were detected as ions *m/z* 1712.12 and 1835.13. The *m/z* difference of 123.01 corresponds to addition of a phosphoethanolamine (PEtN) residue. (B) K. kingae lipid A fatty acid content shown as a percentage of total. Lauric and myristic methyl esters are shown as 12:0 and 14:0, respectively. Hydroxylated methyl esters at the 3-position are denoted with 3-OH. (C and D) Key fragment ions and corresponding structures identified by FLAT^n^ and MS/MS for K. kingae lipid A species detected at ion *m/z* 1712.12 (C) and the PEtN-modified lipid A detected at ion *m/z* 1835.13 (D). Download FIG S5, TIF file, 2.8 MB. Copyright © 2022 Montoya et al. 2022 Montoya et al. https://creativecommons.org/licenses/by/4.0/ This content is distributed under the terms of the Creative Commons Attribution 4.0 International license.

Gas chromatography-flame ionization detection analysis of fatty acid content revealed four fatty acids attributed to the lipid A structure. The 12:0(3-OH) and 14:0(3-OH) fatty acids represent the fatty acids that are ester and amide linked to the glucosamine backbone, respectively. The 12:0 fatty acid represents the fatty acids that are ester linked to the hydroxyl group at the 3-position. There is a small population of 14:0 fatty acids that are likely in place of 12:0 fatty acids, a common occurrence seen across several lipid A structures (see Fig. S5B). These parallel characterization approaches confirmed the structure of the K. kingae lipid A and provided additional evidence that the HMW LPS is composed of LMW LPS with a galactan modification.

### The *pamDE* genes are necessary to promote bacterial resistance to polymyxin B-mediated killing.

**FIG 6:**
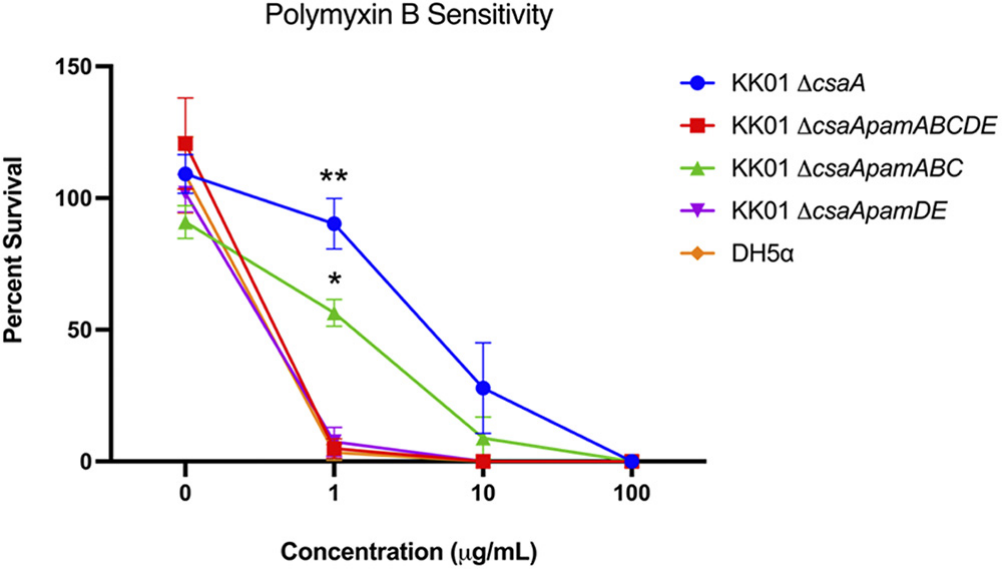
KK01 Δ*csaApamDE* mutants are susceptible to killing by polymyxin B. K. kingae strains KK01 Δ*csaA*, Δ*csaApamABCDE*, Δ*csaApamABC*, and Δ*csaApamDE* were challenged with various concentrations of polymyxin B for 30 min. CFU were enumerated and survival was determined as a percentage of the inoculum. Data are presented as means ± SEM from three independent experiments. Statistical significance was determined relative to strain KK01 Δ*csaApamDE* by 2-way analysis of variance ANOVA with Tukey’s correction for multiple comparisons. *, *P* < 0.05; **, *P* < 0.01.

To evaluate the independent functional roles of LPS and galactan, we performed bactericidal assays with polymyxin B over a range of concentrations. Polymyxin B is a cationic antimicrobial peptide that works by displacing the LPS-coordinated metal ions. Previous work by Muñoz et al. attributed polymyxin B resistance in K. kingae to galactan, prior to our knowledge that surface expression of galactan may be dependent on the presence of LMW LPS (8). To understand the relative contributions of LPS and galactan to polymyxin B sensitivity, we performed survival assays with strains KK01 Δ*csaA*, KK01 Δ*csaApamABCDE*, KK01 Δ*csaApamABC*, and KK01 Δ*csaApamDE*. As shown in Fig. 6, survival of strain KK01 Δ*csaApamABC* was not significantly decreased by polymyxin B relative to that of the parent KK01 Δ*csaA*. However, survival of strains KK01 Δ*csaApamABCDE* and KK01 Δ*csaApamDE* was markedly decreased in the presence of polymyxin B (Fig. 6). These results establish that the *pamDE* genes are critical for survival in polymyxin B, indicating the key role of LMW LPS.

## DISCUSSION

**FIG 7:**
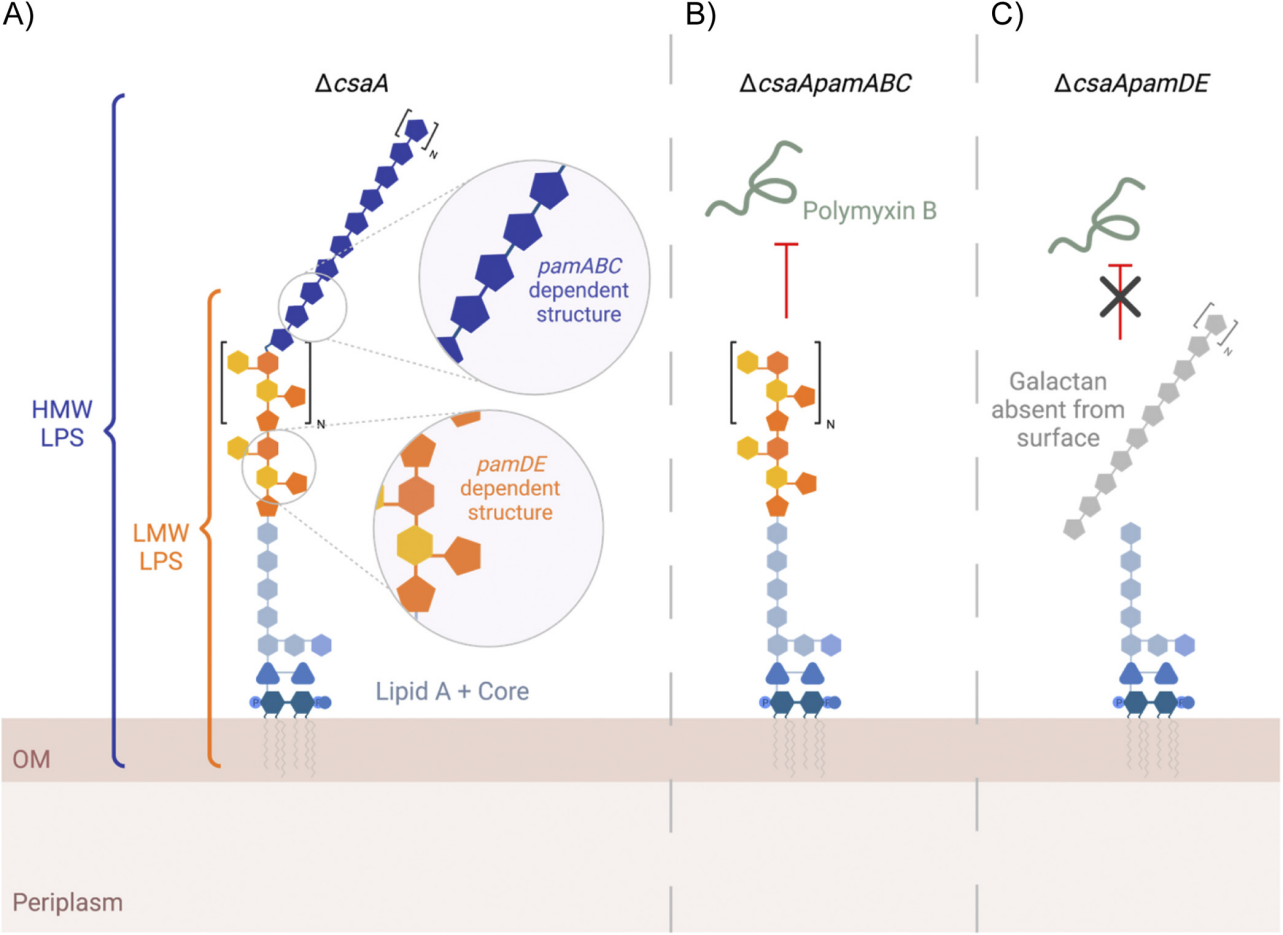
Summary of the relationship between the K. kingae galactan and lipopolysaccharide. Proposed models of the multiple LPS glycoforms expressed by strains KK01 Δ*csaA* (A), KK01Δ *csaApamABC* (B), and KK01 Δ*csaApamDE* (C). The data in this study suggest that LMW LPS glycoforms are composed of lipid A, core oligosaccharide, and the *pamDE*-dependent atypical O-antigen, and HMW LPS glycoforms are composed of lipid A, core oligosaccharide, and the *pamABC*-dependent galactan. Note that the orange shaded O-antigen is depicted as a general repeating unit, because the structure is currently unknown. Expression of the *pamDE*-dependent structure is required for inhibition of polymyxin B-mediated killing.

Kingella kingae is the leading cause of osteoarticular infections in young children and expresses a collection of surface polysaccharides that contribute to the pathogenesis of disease. The galactan promotes both resistance to serum complement and evasion of neutrophil killing (8). Galactan is encoded by the *pamABC* genes, which are present in the five-gene *pamABCDE* cluster. In this study, we showed that the *pamD* and *pamE* genes in this cluster are required for production of an atypical O-antigen and encode proteins with a predicted glycosyltransferase fold, suggesting that they encode LPS glycosyltransferases. Further analysis by DOC-PAGE and NMR revealed that wild-type K. kingae produced multiple glycoforms of LPS. They included LMW LPS composed primarily of lipid A, core oligosaccharide, and O-antigen with a repeating unit, and a discrete HMW LPS composed of lipid A, core oligosaccharide, O-antigen, and galactan (Fig. 7). Additional analysis demonstrated that presence of galactan on the bacterial surface is dependent on anchoring to the atypical O-antigen, which in turn is the primary determinant of K. kingae resistance to polymyxin B.

NMR and compositional analyses on fractionated LPS and O-PS released from lipid A established that HMW LPS contains a large portion of galactofuranose, suggesting the presence of a novel covalent linkage between the K. kingae LPS and galactan. In support of this possibility, we showed that galactan can be detected with a galactan-specific antiserum (GP-19) in an LPS preparation from bacteria with an intact *pam* locus. The galactan used for generation of the galactan-specific antiserum was isolated from the bacterial surface and purified by size exclusion chromatography, making it unlikely that the copurifying LPS was a contaminant. Fractionation and structural analyses of the HMW LPS and LMW LPS fractions allowed us to identify K. kingae lipid A signatures in both fractions, further supporting that the copurification of LPS and galactan is the result of anchoring of galactan to the atypical O-antigen in LMW LPS. It is important to note that a direct covalent linkage between galactan and LMW LPS could not be observed by NMR because of the complexity of the LMW LPS fraction and possible lability of the Gal*f* linkage during release of the O-PS by acid hydrolysis. However, the biological data and the preliminary NMR analysis presented in this work provide compelling evidence that a covalent linkage exists, uncovering a novel physical relationship between a bacterial exopolysaccharide and LPS. Based on this evidence, the K. kingae galactan exopolysaccharide may be viewed as an unusual modification of the O-antigen, as opposed to a traditional secreted exopolysaccharide.

In other organisms, modifications to the O-antigen contribute to the heterogeneity of LPS structures and are recognized as an important driver of host immune recognition (31–33). Modification of the O-antigen with acetyl or fucosyl groups is characterized by the transfer of single moieties to individual repeating units and typically occurs in the periplasm by transferase genes present in the major O-antigen gene cluster (34–36). Currently, there are no other examples of a bacterial LPS with an O-antigen-like structure that is further modified with a very large homopolymeric sugar component. While our data suggest that the transferase required for attachment may be encoded by the *pam* locus, more studies are needed to determine which genes are immediately responsible for creating a covalent linkage between galactan and LMW LPS and to identify the point of attachment.

There are few examples of an association between any component of LPS and an exopolysaccharide. The closest parallels to our observation with K. kingae are the expression of M-antigen in E. coli K-12 and the ECA_LPS_ in Yersinia enterocolitica. M-antigen is an LPS glycoform in which colanic acid (CA) exopolysaccharide repeat units are polymerized onto the growing LPS molecule with a ligation point at the O-7 of the l-glycero-d-mannose-heptose in the LPS outer core region (14). This is the same residue to which the O-antigen is attached; thus, under CA-inducing conditions, a portion of the O-antigen is replaced with CA. While there is some evidence to suggest that ECA_LPS_ and O-antigen can coexist in the same LPS molecule, the attachment point has been shown to be housed in the inner core oligosaccharide, suggesting that both components can be attached independently of one another (37). Conversely, our data indicate that expression of *pamDE* and a complete LMW LPS O-antigen is required for surface presence of galactan, suggesting that in K. kingae the O-antigen and galactan are components of the same LPS molecule and that galactan anchoring is dependent on expression of the complete O-antigen.

The LPS genetic machinery is often a good indicator of the mechanism of O-antigen chain length regulation. It is likely that more rigorous genetic mining will be required to identify chain length regulation genes in K. kingae. However, the LPS fractionation presented in this work revealed the presence of two isolated modal clusters of LPS species, suggesting that both the HMW LPS and LMW LPS have a narrow distribution of chain lengths (38). O-antigen chain length regulation largely occurs by one of two pathways: the ABC transporter-dependent pathway or the *wzx/wzy* pathway (39). In E. coli and S. enterica, the ABC transporter-dependent pathway has been associated with homopolymeric O-antigens (40, 41). Similarly, in Pseudomonas aeruginosa, which expresses multiple glycoforms of LPS simultaneously, the ABC transporter-dependent pathway is required for export and regulation of the homopolymeric common polysaccharide antigen (42–44). This pathway begins with initial synthesis of a sugar residue on a lipid adapter on the cytoplasmic leaf of the inner membrane. Once a complete polymer is assembled, it is flipped into the periplasm by the activity of the *wzm/wzt* ABC transporter (39, 40, 45). Once in the periplasm, the O-antigen homopolymer is ligated to the lipid A-core oligosaccharide by the integral membrane protein WaaL (40). Recently, we identified a putative *waaL* homolog in K. kingae, and future analyses will determine if expression of this gene is required for production of HMW LPS or LMW LPS.

Given our current understanding of the homopolymeric nature of galactan and the lack of obvious *wzx/wzy* homologs in the K. kingae genome, we hypothesize that HMW LPS may be regulated in an ABC transporter-dependent manner. Preliminary data suggest that the *pamABCDE* gene products lack sequence signals for export, indicating that they are likely located in the cytoplasm. These observations together with the predicted functions of the gene products support a model that involves cytoplasmic assembly of the complete O-antigen, galactan, and ABC transporter-dependent delivery into the periplasm. Future experiments are required to identify membrane-bound O-antigen biosynthesis components and evaluate the individual functions of the *pamABCDE* gene products in the process. As an additional consideration, it is possible that the K. kingae LPS requires two independent mechanisms of chain regulation for the HMW LPS and LMW LPS glycoforms. The chain length of LMW LPS may be regulated in a substrate-mediated manner. In other words, the addition of a critical residue may trigger the addition of the complete galactan polymer, constraining the distribution of chain lengths of LMW LPS. More mutational analyses are required to determine the substrate preference of the *pam* glycosyltransferases. Coupling of this information with detailed compositional and structural analyses of LMW LPS may provide insight into the mechanism of chain length regulation of the K. kingae LPS.

Much work has been done to characterize the function of galactan. Initial studies performed by Bendaoud et al. showed that galactan exhibited widespread antibiofilm properties against biofilms of *Kingella* and other genetically diverse bacteria (10). More recently, our group examined the role of galactan in resistance to immune mechanisms. These studies demonstrated that galactan is sufficient to prevent complement-mediated killing in the absence of the polysaccharide capsule (5), in contrast with observations in other encapsulated organisms, where the capsule plays a dominant role in serum resistance and exopolysaccharides have rarely been implicated (46–48). In addition to the unique role of galactan in serum resistance, we found that galactan inhibits neutrophil phagocytosis (8). We also reported that galactan mediates resistance to killing by cationic antimicrobial peptides (CAMPs) (8). It is noteworthy that all previous characterizations of galactan have been performed in the context of a full *pamABCDE* locus deletion. In this study, we showed that the deletion of *pamDE* results in the loss of both LMW LPS and surface expression of galactan, a confounding factor that was not considered in earlier work. Our results in this study established that the Δ*csaApamDE* mutant was significantly more susceptible than the Δ*csaA* parent to polymyxin B killing, while the Δ*csaApamABC* mutant displayed a minor increase in susceptibility that was not statistically significant. CAMPs such as polymyxins function through electrostatic interactions with the Gram-negative bacterial outer membrane, which is made negatively charged by the phosphate backbone of the LPS (49, 50). Substitution of lipid A with positively charged moieties like PEtN and 4-amino-4-deoxy-l-arabinose (l-Ara4N) decreases the net negative charge of the membrane and weakens interactions between polymyxins and the membrane, inhibiting bacterial killing (51, 52).

As an interesting comparison, in Burkholderia cenocepacia a complete inner core oligosaccharide is required for resistance to polymyxins (52). In addition to providing the foundation for modifications that increase the net positive charge of the membrane, the inner core oligosaccharide is integral for membrane integrity, which is a prerequisite for antimicrobial resistance (53). Here, we have shown that the K. kingae lipid A is modified with PEtN, which may contribute to polymyxin B resistance, as has been described in the literature (51, 54, 55). Additionally, our data suggest that expression of *pamDE* and thus a complete LMW LPS is required for maximal polymyxin B resistance. More work is needed to determine if the mechanism of resistance is through modification of the LPS, steric occlusion of the lipid A backbone by the O-antigen, or crucial membrane integrity that is maintained by LMW LPS. Overall, our data suggest that resistance to CAMPs is primarily mediated by LMW LPS, with little influence of galactan. These results have begun to challenge our understanding of the function of both LPS and galactan, specifically, the processes in which these polysaccharides function jointly versus independently.

While this work was performed with our prototype strain KK01 expressing a →5)-β-Gal*f*-(1→ exopolysaccharide, a second exopolysaccharide with the structure →3)-β-Gal*f*-(1→6)-β-Gal*f*-(1→ has been identified in other clinical isolates of K. kingae (5, 9, 10). In clinical strain PYKK181, the →3)-β-Gal*f*-(1→6)-β-Gal*f*-(1→ galactan has been shown to promote biofilm dispersal (10). However, this exopolysaccharide has not yet been characterized with regard to resistance to serum and neutrophils. Similarly, the diversity of LPS across K. kingae isolates is unknown.

Earlier work characterized the K. kingae galactan and identified the biosynthetic machinery encoded by the *pam* locus (7, 9). In this work, we showed that the *pamD* and *pamE* genes encode LPS glycosyltransferases that are required for expression of K. kingae LMW LPS and for surface presence of galactan. LPS fractionation and NMR revealed that K. kingae HMW LPS contains lipid A, core oligosaccharide, an O-antigen, and galactan, suggesting that galactan is anchored to LMW LPS and revealing novel functions for the *pam* genes. This is the first description of an interaction between K. kingae LPS and galactan, highlighting the unique structure of these surface polysaccharides and providing insight into their functions in the pathogenesis of K. kingae disease.

## MATERIALS AND METHODS

### Bacterial strains and growth conditions.

The strains used in this study are listed in Table S3 in the supplemental material. K. kingae and H. influenzae strains were stored at −80°C in brain heart infusion (BHI) broth with 20% glycerol. Escherichia coli strains were stored at −80°C in Luria-Bertani (LB) broth with 15% glycerol. K. kingae strains were grown at 37°C with 5% CO_2_ on chocolate agar. E. coli strains were grown at 37°C on LB agar or shaking at 250 rpm in LB broth supplemented with 100 μg/mL ampicillin or 50 μg/mL kanamycin, as appropriate.

10.1128/mbio.02295-22.10 TABLE S3 Bacterial strains and plasmids used in this study. Download Table S3, DOCX file, 0.03 MB. Copyright © 2022 Montoya et al. 2022 Montoya et al. https://creativecommons.org/licenses/by/4.0/ This content is distributed under the terms of the Creative Commons Attribution 4.0 International license.

10.1128/mbio.02295-22.10 TABLE S4 Primers used in this study. Download Table S4, DOCX file, 0.01 MB. Copyright © 2022 Montoya et al. 2022 Montoya et al. https://creativecommons.org/licenses/by/4.0/ This content is distributed under the terms of the Creative Commons Attribution 4.0 International license.

### K. kingae mutant strain construction.

Briefly, plasmid-based gene deletion constructs were created in E. coli, linearized, and introduced into K. kingae using natural transformation (6). Transformants were recovered by selectively plating on chocolate agar plates containing 50 μg/mL kanamycin or 1 μg/mL erythromycin, as appropriate. The introduced mutations were confirmed by PCR and Sanger sequencing. The primers used for mutant strain construction are listed in Table S4. The detailed methods for generating the K. kingae mutants used in this study are described in Text S1 in the supplemental material.

10.1128/mbio.02295-22.2 TEXT S1 Supplementary methods and materials. Download Text S1, DOCX file, 0.03 MB. Copyright © 2022 Montoya et al. 2022 Montoya et al. https://creativecommons.org/licenses/by/4.0/ This content is distributed under the terms of the Creative Commons Attribution 4.0 International license.

### Surface wash extracts.

Bacteria were cultured for 16 to 20 h and suspended in 5 mL PBS to an optical density at 600 nm (OD_600_) of 0.8. After 30 min of gentle agitation at ambient temperature, the bacteria were removed by centrifugation (30 min at 3,220 × *g*), and the supernatant was filtered through a 0.22-μm filter. The resulting supernatant was concentrated to approximately 250 μL over a 50,000-molecular weight cutoff (MWCO) Amicon Ultra centrifugal filter (MilliporeSigma, Burlington, MA, USA). The samples were supplemented with 2.5 mM MgCl_2_ and 0.1 mM CaCl_2_, treated with 1 U of DNase I and 10 μg of RNase A for 6 h at 37°C, and then treated with 20 μg of proteinase K for 16 h at 50°C, prior to analysis by silver staining and Western blotting.

### LPS isolation for DOC-PAGE and ELISA.

To isolate LPS, the bacterial strains were grown on chocolate agar for 16 to 20 h and resuspended in BHI for the preparation of bacterial lawns. Lawns were grown 16 to 20 h on chocolate agar, and the lawns from five plates were pooled by resuspending in PBS. After centrifugation, the pellets were then resuspended in equal amounts of 90% phenol and endotoxin-free water, incubated rotating at 70°C for 1 h, and then centrifuged at 10,000 rpm for 10 min to separate the phenolic and aqueous phases. The aqueous phase containing LPS was saved. The phenol layer was reextracted with 500 μL endotoxin-free water two more times, and the aqueous layers were pooled. The remaining residual phenol was removed by two 2-mL washes with diethyl ether. The aqueous samples were lyophilized overnight. The lyophilized LPS pellets were resuspended in 1 mL of endotoxin-free water and stored at −20°C. To eliminate contaminating nucleic acids and proteins, LPS samples were treated with 2 U DNase I and 100 μg/mL RNase A for 6 h at 37°C and 100 μg/mL proteinase K at 45°C for 16 to 20 h.

For ELISA and Western blot analyses, LPS amounts were standardized by Kdo content as described previously (56). Briefly, lyophilized LPS samples were resuspended in endotoxin-free water. To a 50-μL sample, an equal volume of 0.5 N sulfuric acid was added, and the samples were heated at 100°C for 20 min. The samples were treated with 36 mM periodate, incubated at room temperature for 10 min, and then treated with 0.2 M sodium arsenite and 29 mM thiobarbituric acid. The samples were heated for 8 min, cooled to room temperature, and then treated with 1.5 mL of butanol reagent (95% butanol, 5% concentrated HCl). Following centrifugation to separate the phases, the upper butanol layer was removed to a cuvette and promptly read on a spectrophotometer at wavelengths of 509 nm and 552 nm. The difference between the two readings was calculated and compared to a Kdo standard curve.

### LPS isolation for size exclusion chromatography and NMR.

The harvested bacterial cells were resuspended in PBS, and the cell suspension was centrifuged for 20 min at 5,000 × *g* at 4°C. The pellets were washed four times until deprived of a viscous supernatant. The crude LPS was obtained from the water phase by applying the hot phenol-water extraction method described by Westphal and Ian (57). The water phase was dialyzed against distilled H_2_O (14,000 MWCO dialysis membrane) and then freeze-dried. Nucleic acids and proteins were removed by 12-h treatment with RNase A and DNase I at 37°C, followed by 12 h of incubation with proteinase K at 45°C and dialysis (14,000 MWCO, 4°C) against several exchanges of H_2_O. Finally, the samples were ultracentrifuged at 100,000 × *g* at 4°C for 18 h. The LPS pellets were resuspended in water, freeze-dried, and extracted with 9:1 (vol/vol) ethanol in water at 4°C to remove traces of phospholipids.

### Composition analysis of LPS.

The glycosyl composition of LPS was determined by preparation of trimethylsilyl (TMS) methyl glycosides after 18 h of methanolysis of 300 μg of LPS with 1 M HCl-methanol at 80°C, in the presence of an internal standard of *myo*-inositol (20 μg) (58). The TMS method also identified straight-chain and hydroxylated fatty acids constituting LPS (fatty acid methyl esters [FAME] and TMS-FAME, respectively). The TMS and FAME derivatives were analyzed on a Hewlett-Packard HP5890 gas chromatograph equipped with mass selective detector 5970 MSD using an EC-1 fused silica capillary column (30 m, 0.25-mm inner diameter). The oven temperature was 80°C for 2 min, then ramped to 160°C at 20°C/min and to 200°C at 2°C/min, followed by an increase to 250°C at 10°C/min with an 11-min hold.

### DOC-PAGE and silver stain analysis.

The LPS was analyzed by PAGE using acrylamide gels with deoxycholic acid (DOC) in the running buffer. The DOC-PAGE gels were fixed overnight in 40% ethanol and 5% acetic acid and stained with silver after oxidation with sodium periodate (59) or with alcian blue followed by classical silver staining as previously described (60, 61). The combined alcian blue-silver staining method was used to track and evaluate the LPS elution in the dissociative condition size exclusion chromatography in the presence of DOC.

### Generation of GP-19 antigalactan antiserum.

The galactan exopolysaccharide and a recombinant mutant diphtheria toxin carrier protein were purified and conjugated as described in Text S1. The resulting glycoconjugate was sent to Cocalico Biologicals (Stevens, PA) for injection into guinea pig GP-19, using the Ribi adjuvant according to their standard polyclonal antibody production protocol (Cocalico Biologicals IACUC approved project number 2018-0984), generating the GP-19 antiserum.

### Western blotting.

Surface wash extracts, LPS preparations, and digested whole-cell sonicates were each separated according to the DOC-PAGE method described above and were subsequently transferred to nitrocellulose. Following blocking in 5% skim milk in PBS (blocking buffer), the blots were incubated with antiserum GP-19 diluted 1:1,000 in blocking buffer with gentle agitation at ambient temperature for 1 h or at 4°C for 16 to 18 h. Following washing with Tris-buffered saline with 0.1% Tween 20 (TBST), the blots were incubated with a 1:5,000 dilution of anti-guinea pig–horseradish peroxidase (αGP-HRP) in blocking buffer. After washing with TBST, the blots were exposed to a chemiluminescent HRP substrate, and the blot images were captured using a GBox Chemi:XT4 system (Syngene, Frederick, MD).

### ELISAs.

For whole K. kingae ELISAs, the strains were cultured 16 to 20 h, the bacterial growth was swabbed into PBS to an OD_600_ of 0.1, and 100 μL was added to wells of a 96-well plain polystyrene plate. For E. coli whole-cell sonicate ELISAs, the digested whole-cell sonicates were diluted 1:20 in PBS, and 100 μL was added to the wells of a 96-well Microlon 200 medium binding plate (Greiner Bio-One, Kremsmünster, Austria). For LPS samples, equivalent quantities were added to 96-well CovaLink plates (Nunc; ThermoFisher Scientific, Waltham, MA, USA) in 100 μL carbonate buffer (100 mM carbonate, pH 9.6). For all sample types, the plates were processed using the same protocol. Coating was carried out at 4°C for 16 to 20 h, and the plates were then washed with TBST and blocked overnight in 2% milk in PBS. GP-19 antiserum dilutions in 2% milk in PBS served as the source of primary antibody for 1 h at 37°C. The plates were again washed with TBST and were incubated with αGP-HRP (1:2,000) for 1 h at 37°C. The plates were developed with 3,3′,5,5′-tetramethylbenzidine ELISA peroxidase substrate (Rockland Immunochemical, Limerick, PA), and absorbance was measured on a plate reader at 655 nm.

### Fractionation of LPS and release of O-PS.

HMW LPS was separated from LMW LPS by size exclusion chromatography using a Superdex 75 10/300GL (Cytiva) under dissociative conditions in the presence of 0.25% sodium deoxycholate, pH 9.2 (62). The eluting fractions were monitored with refractive index (Shimadzu RID-10A) and with DOC-PAGE stained with alcian blue and silver (see Text S1 for further details on the DOC-PAGE method). The O-PS (polysaccharide portion of LPS, including core, O-antigen, and galactan) was released from the lipid A by using 1% acetic acid for 1.5 to 2 h at 100°C until the formation of insoluble lipid A precipitate. The lipid A precipitate was removed from the O-PS soluble fraction by initial centrifugation for 25 min at 3,500 × *g*. The pellet was washed by addition of water, resuspension, and centrifugation at 100,000 × *g* for 4 h at 4°C. The soluble O-PS fraction was also ultracentrifuged at 100,000 × *g* for 4 h, 4°C, and the supernatant was freeze-dried. The dry O-PS was dissolved in water and passed through a 0.22-μm nylon filter to remove any trace of free lipids, LPS, or lipid A and was used for structural work.

### NMR analysis.

Isolated LPS or released O-PS samples were exchanged to D_2_O by lyophilization, and 50 nmol of 2,2-dimethyl-2-silapentane-5-sulfonate-D6 (DSS-d6; Cambridge Isotope Laboratories) was added to each sample for chemical shift referencing. NMR data were collected at 25°C on a Varian VNMRS (^1^H, 599.66 MHz) or Bruker Avance III (^1^H, 600.13 MHz) spectrometer, each equipped with a 5-mm cryoprobe. To determine the structure of the released O-PS, 1D ^1^H and 2D correlation spectroscopy (COSY), total correlation spectroscopy (TOCSY), nuclear Overhauser effect spectroscopy (NOESY), HSQC, and heteronuclear multiple-bond correlation (HMBC) spectra were collected. The TOCSY and NOESY spectra were collected with presaturation of the residual water signal, and the HSQC spectrum was acquired with signal multiplicity editing. The mixing times were 70 ms (TOCSY) and 150 ms (NOESY). The homonuclear correlations were collected with ^1^H spectral widths of 4,808 Hz, 180 increments, and 8 to 16 scans per increment. The HSQC and HMBC spectra were collected with spectral widths (^1^H and ^13^C) of 7,184 and 10,556 Hz, 128 increments, and 32 (HSQC) or 96 (HMBC) scans per increment. ^1^H and ^13^C chemical shifts were referenced to the respective DSS signals at 0.0 ppm. The NMR data were analyzed in MestreNova 14.1.1.

### MALDI-TOF MS analysis of lipid A from HMW and LMW LPS.

The lipid A released from HMW LPS and LMW LPS fractions that were separated on a Superdex 75 column was analyzed by MALDI-TOF MS using an Applied Biosystems 4800 proteomics analyzer. The lipid A samples were dissolved in a chloroform-methanol solution (3:1 [vol/vol]) and mixed with 0.5 M 2,4,6-trihydroxyacetophenone matrix in methanol in a 1:1 ratio. A 0.5-μL volume of the final mixture was applied onto a stainless steel target plate, and the spectra were acquired in negative reflector ionization mode ([M-H]^−^).

### Polymyxin B bactericidal assays.

Bacterial survival in polymyxin B was determined as described previously (8). Briefly, K. kingae strains were grown on chocolate agar overnight and resuspended in PBS-G (PBS with 0.1% gelatin). The bacterial suspensions were diluted to a concentration of 4.0 × 10^4^ CFU/mL, 100-μL aliquots were mixed with polymyxin B (Alfa Aesar, Ward Hill, MA) at various physiological concentrations, and samples were incubated at 37°C with 5% CO_2_ for 30 min. The reaction was stopped by adding 9 mM MgCl_2_ prior to plating. Serial dilutions of the reaction mixtures were plated on chocolate agar plates, and the surviving CFU were enumerated following overnight incubation. Percent survival was calculated by dividing the recovered CFU counts by the inoculum CFU counts.

### Statistical analysis.

Statistical analyses were performed with GraphPad Prism software for Mac (version 9.2.1; GraphPad Software, San Diego, CA). A *P* value of <0.05 was considered statistically significant. The specific statistical tests used for each experiment are specified in the relevant figure legend.
