# Supplementary figures and images for: Surface Anchoring of the Kingella kingae Galactan Is Dependent on the Lipopolysaccharide O-Antigen
Source: mBio. 2022 Sep 7;13(5):e02295-22. doi: 10.1128/mbio.02295-22 (PMC9615999; doi:10.1128/mbio.02295-22)

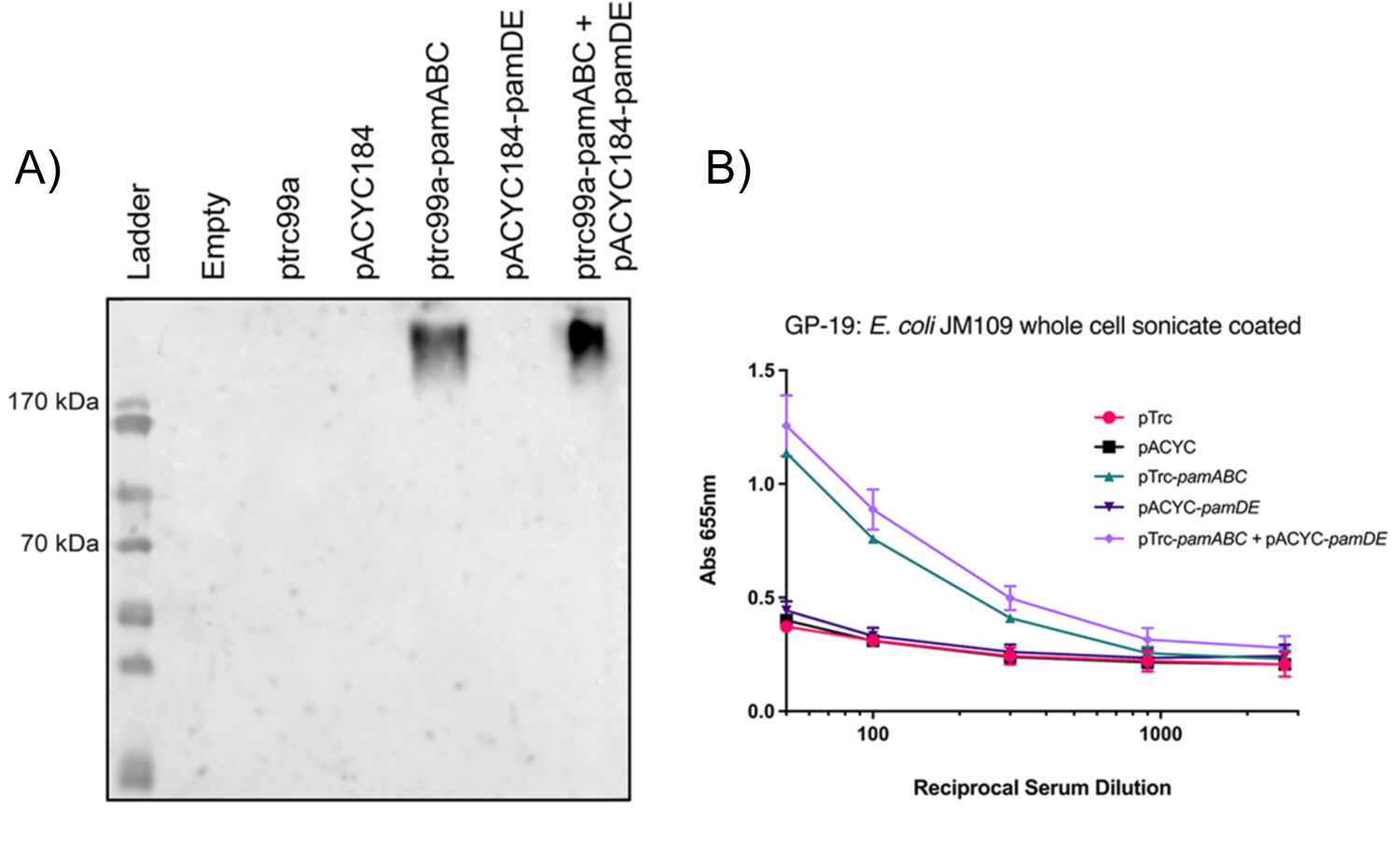

Supplement: FIG S1 [file mbio.02295-22-s0005.tif]

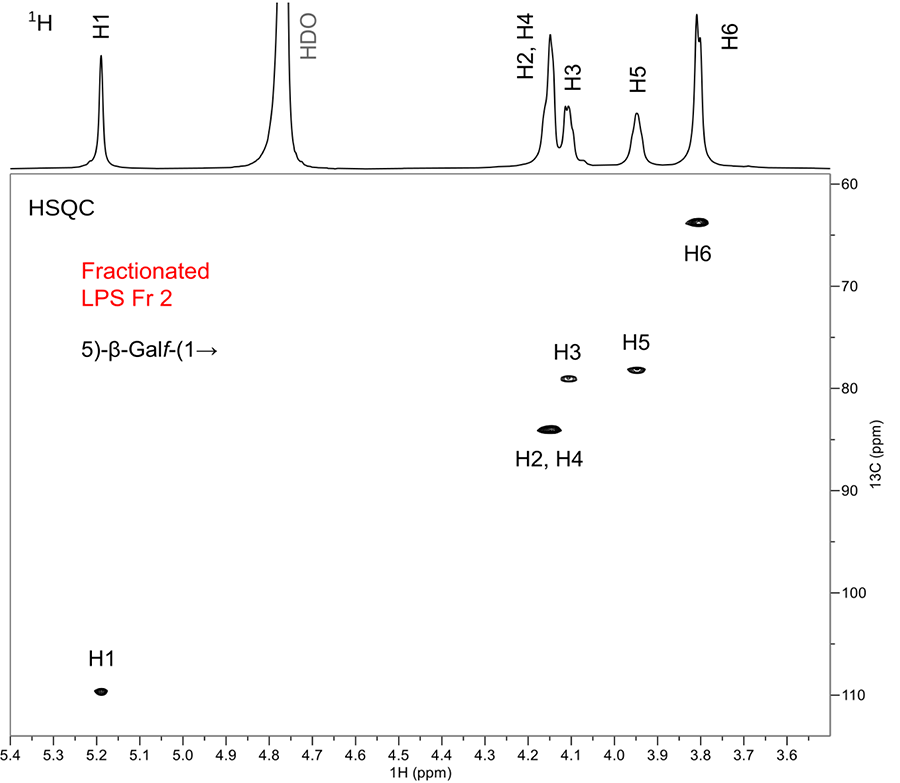

Supplement: FIG S2 [file mbio.02295-22-s0006.tif]

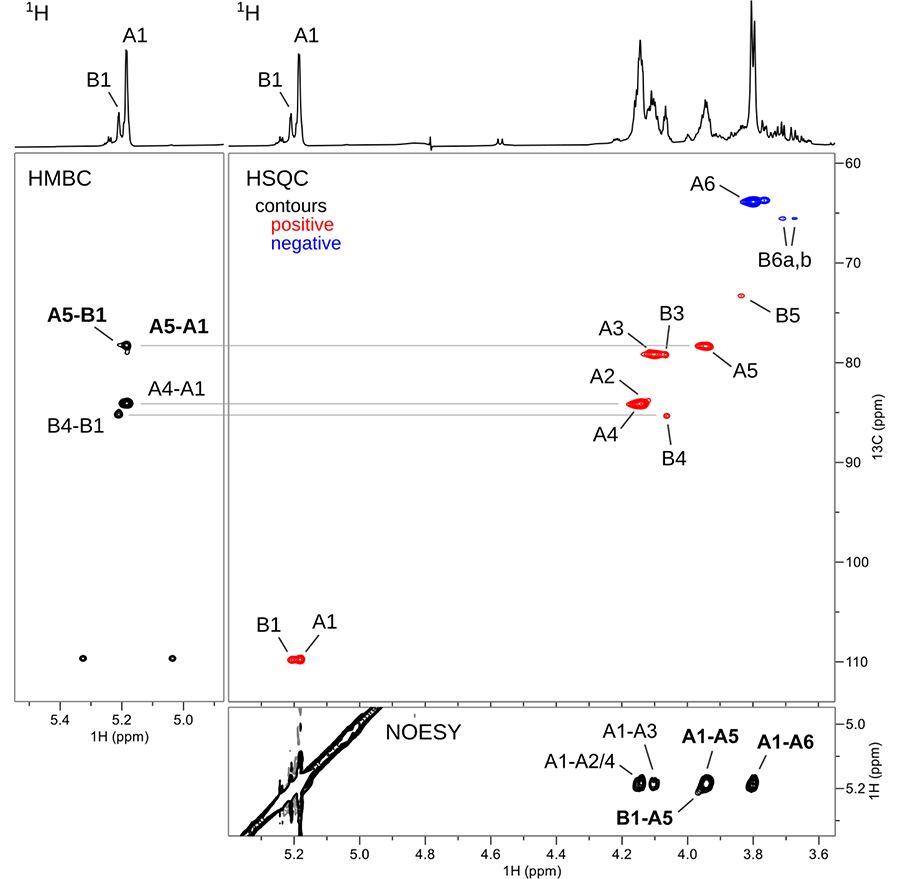

Supplement: FIG S3 [file mbio.02295-22-s0007.tif]

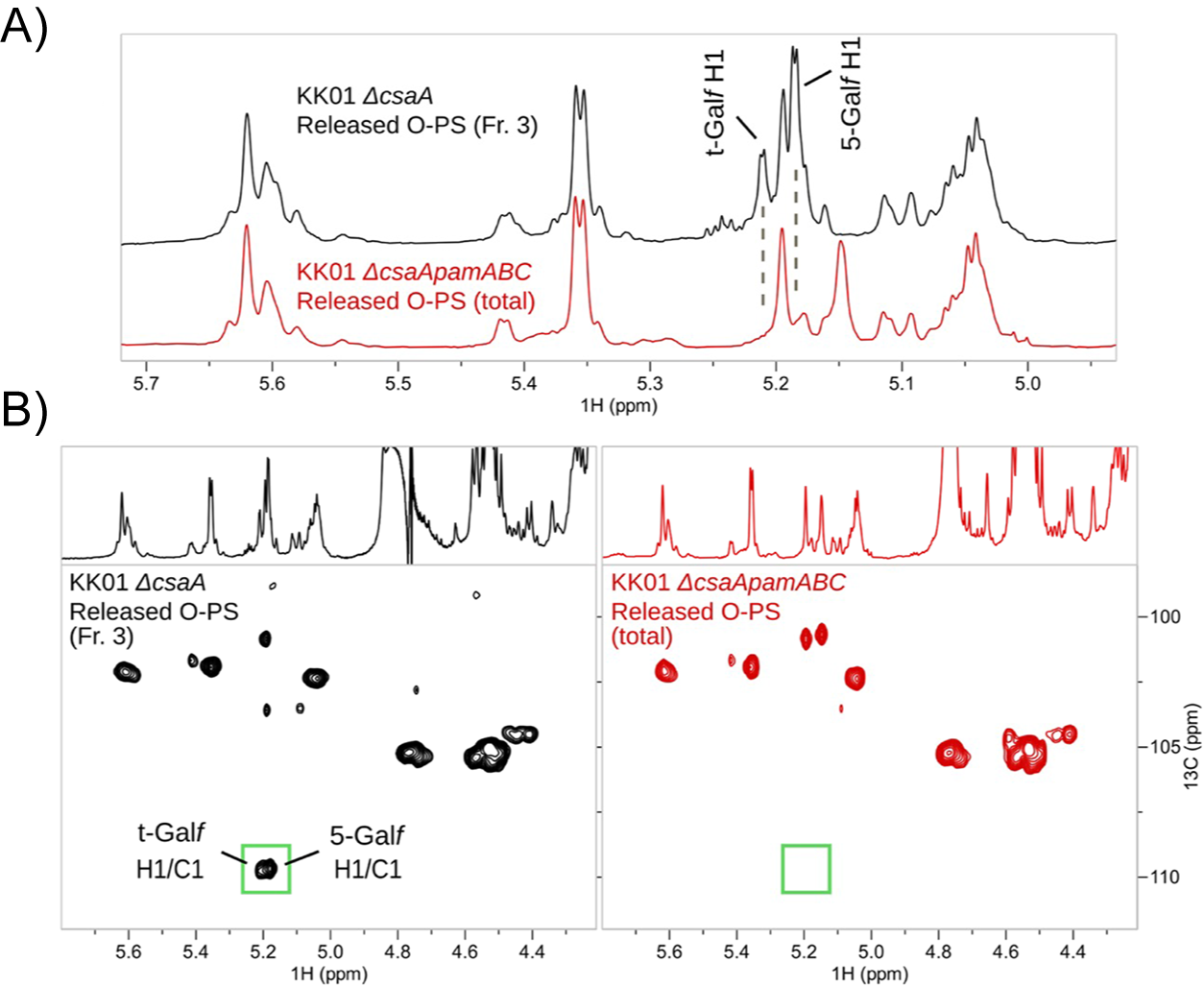

Supplement: FIG S4 [file mbio.02295-22-s0008.tif]

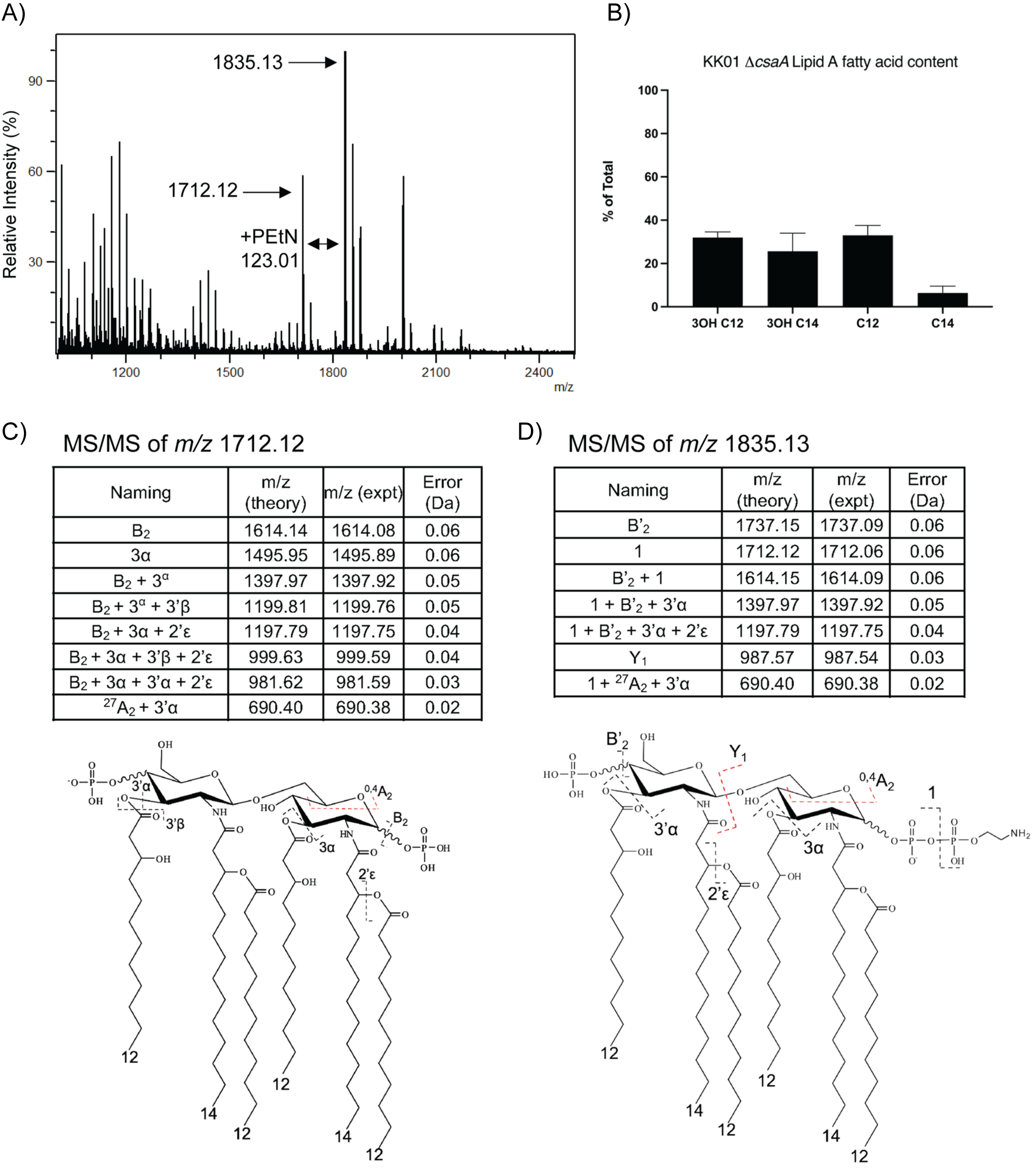

Supplement: FIG S5 [file mbio.02295-22-s0009.tif]
