# Supplementary material for: Surface Anchoring of the Kingella kingae Galactan Is Dependent on the Lipopolysaccharide O-Antigen
Source: mBio. 2022 Sep 7;13(5):e02295-22. doi: 10.1128/mbio.02295-22 (PMC9615999; doi:10.1128/mbio.02295-22)
Supplement: TABLE S1 [file mbio.02295-22-s0001.docx]

| **Residue** | **Mole %** |
| --- | --- |
| Galactose (Gal) | 92.9 |
| Glucose (Glc) | 5.0 |
| N-acetyl Glucosamine (GlcNAc) | 2.1 |
| **Total** | **100.0** |
