# Supplementary material for: Surface Anchoring of the Kingella kingae Galactan Is Dependent on the Lipopolysaccharide O-Antigen
Source: mBio. 2022 Sep 7;13(5):e02295-22. doi: 10.1128/mbio.02295-22 (PMC9615999; doi:10.1128/mbio.02295-22)
Supplement: TABLE S2 [file mbio.02295-22-s0002.docx]

|  | Group | | | | | |
| --- | --- | --- | --- | --- | --- | --- |
| Residue | **1** | **2** | **3** | **4** | **5** | **6(a,b)** |
| **A** | 5.19 | 4.14 | 4.11 | 4.16 | 3.95 | 3.80 |
| 5)-β-Gal*f*-(1→ | 109.6 | 84.0 | 79.0 | 84.1 | 78.2 | 63.7 |
| **B** | 5.22 | 4.15 | 4.07 | 4.07 | 3.84 | 3.72, 3.67 |
| t-β-Gal*f*-(1→ | 109.8 | 83.9 | 79.1 | 85.3 | 73.2 | 65.4 |
|  |  |  |  |  |  |  |
| Linkages | Method | |  |  |  |  |
| A1 → A5 | NOESY, HMBC  NOESY, HMBC | | |  |  |  |
| B1 → A5 |  |  |  |  |  |  |
