# Supplementary material for: Surface Anchoring of the Kingella kingae Galactan Is Dependent on the Lipopolysaccharide O-Antigen
Source: mBio. 2022 Sep 7;13(5):e02295-22. doi: 10.1128/mbio.02295-22 (PMC9615999; doi:10.1128/mbio.02295-22)
Supplement: TABLE S3 [file mbio.02295-22-s0003.docx]

| Strain or Plasmid | Description | Source or Reference |
| --- | --- | --- |
| *K. kingae strains* |  |  |
| KK01 | ﻿Nonspreading/noncorroding derivative of clinical isolate 269–492 | (1) |
| KK01 Δ*csaA* | KK01 *csaA::un,* contains *csaA* deletion | This study |
| KK01 Δ*csaApamABCDE* | KK01 *csaA::un pam::kan,* contains *csaA* deletion and *pam* locus deletion | This study |
| KK01 Δ*csaApamABC* | KK01 *csaA::un pamABC::kan,* contains *csaA* deletion and *pamABC* deletion | This study |
| KK01 Δ*csaApamDE* | KK01 *csaA::un pamDE::kan,* contains *csaA* deletion and *pamDE* deletion | This study |
| KK01 Δ*csaApamABCrfaF* | KK01 *csaA::un pamABC::kan rfaF::erm,* contains *csaA* deletion, *pamABC* deletion and *rfaF* deletion | This study |
| *H. influenzae strains* |  |  |
| Rd | Non-adherent lab strain of *H. influenzae,* capsule-deficient serotype D | (2, 3) |
| Rd Δ*rfaF* | Rd *rfaF::kan,* contains *rfaF* deletion | (3, 4) |
| *E. coli strains* |  |  |
| DH5α | ﻿$\lambda$^—^$\phi$80d*lac*ZΔM15 Δ(*lacZYA-argF*)*U169* *recA1 endA1* hsdR17(r_K_^—^ m_K_^—^) *supE44 thi-1* *gyrA relA1* | ThermoFisher Scientific |
| JM109 | *end*A1, *rec*A1, *gyr*A96, *thi, hsd*R17 (r_k_^–^, m_k_^+^), *rel*A1, *sup*E44, Δ( *lac-pro*AB), [F´ *tra*D36, *pro*AB, *laq*I^q^ZΔM15]. | Promega |
| *Plasmids* |  |  |
| pTrc99a | High copy number expression vector containing the Trc promoter | (5) |
| pACYC184 | Low copy number plasmid vector containing the replication system of miniplasmid p15A | (6) |
| pUC19 | High copy number cloning vector | (7) |
| pFalcon2 | Source of the *aphA3* cassette | (8) |
| pIDN4 | Source of the *ermC* cassette | (9) |
| pUC19*csaA*::unmarked | Construct for introduction of an unmarked *csaA* deletion in *K. kingae* | (10) |
| pUC19*pam*::*ermC* | Construct for introduction of an *ermC-*marked *pamABCDE* deletion in *K. kingae* | (11) |
| pTrc99a-*pamABC* | pTrc99a containing *pamABC* under control of an IPTG-inducible promoter | This study |
| pACYC184-*pamDE* | pACYC184 containing *pamDE* under control of the native *pamD* promoter | This study |
| pΔ*pamABC* | pUC19-based plasmid for introduction of the *aphA3*-marked Δp*amABC* deletion | This study |
| pΔ*pamDE* | pUC19-based plasmid for introduction of the *aphA3*-marked Δ*pamDE* deletion | This study |
| pΔ*rfaF* | pUC19-based plasmid for introduction of the *ermC*-marked Δ*rfaF* deletion | This study |

**Supplemental references**

1. 1. Kehl-Fie TE, St. Geme JW. 2007. Identification and characterization of an RTX toxin in the emerging pathogen Kingella kingae. J Bacteriol 189:430–436.

2. Setlow JK, Randolph ML, Boling ME, Mattingly A, Price G, Gordon MP. 1968. Repair of DNA in Haemophilus Influenzae. II. Excision, Repair of Single-Strand Breaks, Defects in Transformation, and Host Cell Modification in UV-Sensitive Mutants. Cold Spring Harb Symp Quant Biol 33:209–218.

3. Spahich NA, Hood DW, Moxon ER, St Geme JW. 2012. Inactivation of Haemophilus influenzae lipopolysaccharide biosynthesis genes interferes with outer membrane localization of the hap autotransporter. J Bacteriol 194:1815–22.

4. Nichols WA, Gibson BW, Melaugh W, Lee NAG, Sunshine M, Apicella MA. 1997. Identification of the ADP-L-glycero-D-manno-heptose-6-epimerase (rfaD) and heptosyltransferase II (rfaF) biosynthesis genes from nontypeable Haemophilus influenzae 2019. Infect Immun 65:1377.

5. Amann E, Ochs B, Abel KJ. 1988. Tightly regulated tac promoter vectors useful for the expression of unfused and fused proteins in Escherichia coli. Gene 69:301–315.

6. Chang ACY, Cohen SN. 1978. Construction and Characterization of Amplifiable Multicopy DNA Cloning Vehicles Derived from the P15A Cryptic Miniplasmid 134:1141–1156.

7. Yanisch-Perron C, Vieira J, Messing J. 1985. Improved M13 phage cloning vectors and host strains: nucleotide sequences of the M13mpl8 and pUC19 vectors. Gene 33:103–119.

8. Hendrixson DR, Akerley BJ, DiRita VJ. 2001. Transposon mutagenesis of Campylobacter jejuni identifies a bipartite energy taxis system required for motility. Mol Microbiol 40:214–224.

9. Hamilton HL, Schwartz KJ, Dillard JP. 2001. Insertion-Duplication Mutagenesis of Neisseria: Use in Characterization of DNA Transfer Genes in the Gonococcal Genetic Island. J Bacteriol 183:4718.

10. Starr KF, Porsch EA, Seed PC, St Geme III JW. 2016. Genetic and Molecular Basis of Kingella kingae Encapsulation https://doi.org/10.1128/IAI.00128-16.

11. Starr KF, Porsch EA, Heiss C, Black I, Azadi P, St. Geme JW. 2013. Characterization of the Kingella kingae Polysaccharide Capsule and Exopolysaccharide. PLoS One 8:e75409.
