# Supplementary material for: Surface Anchoring of the Kingella kingae Galactan Is Dependent on the Lipopolysaccharide O-Antigen
Source: mBio. 2022 Sep 7;13(5):e02295-22. doi: 10.1128/mbio.02295-22 (PMC9615999; doi:10.1128/mbio.02295-22)
Supplement: TABLE S4 [file mbio.02295-22-s0004.docx]

| Primer Name | Sequence 5’ 🡪 3’ |
| --- | --- |
| *pamA*5’_F | GCGAATTCGGCGTTGGTGGAATATCCTG |
| *pamA*5'_R | ACGTGGTACCACCTTCTGGTCGCTGAAATG |
| *pamC*3’_F | ACGTGGATCCCTATGTCGCAACGTTTGATAGG |
| *pamC*3’_R | ACGTAAGCTTGAATGTTGTCCAGCGCAATC |
| *aphA3*_F | GCATGGTACCCATCTAAATCTAGGTACTAAAACAATTCATCCAG |
| *aphA3*_R | GCATGGATCCGTTTGACAGCTTATCATCGATAAACCCAG |
| *pamD*5'_F | ACGTGAATTCTCGTGCTTATGCGCTGTTGC |
| *pamD*5’_R | ACGTGGTACCTTTCGGGATATTGCGGTTGG |
| *pamE*3’_F | GCGGATCCTCAAAGGCTGGTATAAACAC |
| *pamE*3’_R | GCAAGCTTCCATATCGCTTTGGCTTTGC |
| *rfaF5’*_F | ACGTGAATTCGGGAACAAGATTGCTGTAAC |
| *rfaF5’*_R | ACGTGGTACCCAATCCACGATGGGGAAATG |
| *rfaF3*’_F | ACGTGGATCCGCTTGCTGGACGAATTAAAC |
| *rfaF3’*_R | ACGTAAGCTTTAACGTGTCCCACAGGAATC |
| *ermC*_F | ACGTGGATCCGGTTACGCTTTGGGGAAATTATGAGG |
| *ermC*_R | ACGTGGTACCGTAATCATGGTCATAGCTGTTCGATAAGC |
| *pamABC*_F | AGCTGAATTCATGTTCCAATTAAGCGAAATTCC |
| *pamABC*_R | ACGTGGTACCTTTCGGGATATTGCGGTTGG |
| *pamDE*_F | GCGACCACACCCGTCCTGTGCTATGTCGCAACGTTTGATAG |
| *pamDE*_R | AAGGCTCTCAAGGGCATCGGACATTATTTAAATCCCAAATAATTCATAG |
